# A Label-free Multicolor Optical Surface Tomography (ALMOST) imaging method for nontransparent 3D samples

**DOI:** 10.1186/s12915-018-0614-4

**Published:** 2019-01-07

**Authors:** Axelle Kerstens, Nikky Corthout, Benjamin Pavie, Zengjin Huang, Frank Vernaillen, Greetje Vande Velde, Sebastian Munck

**Affiliations:** 1 VIB Bio Imaging Core, Herestraat 49, Box 602, 3000 Leuven, Belgium; 2 Research Group Molecular Neurobiology, Department of Neuroscience, KU Leuven, Herestraat 49, Box 602, 3000 Leuven, Belgium; 3 VIB Center for Brain and Disease Research, KU Leuven, Herestraat 49, Box 602, 3000 Leuven, Belgium; 4 Neuronal Wiring Lab, Department of Neuroscience, KU Leuven, Herestraat 49, Box 602, 3000 Leuven, Belgium; 5 VIB BioInformatics Core, Rijvisschestraat 126 3R, 9052 Ghent, Belgium; 6 Department of Imaging and Pathology, KU Leuven - University of Leuven, Herestraat 49, Box 505, 3000 Leuven, Belgium

**Keywords:** 3D imaging, Tomography, Label-free, Morphology, Real color, Mesoscale, Opaque samples

## Abstract

**Background:**

Current mesoscale 3D imaging techniques are limited to transparent or cleared samples or require the use of X-rays. This is a severe limitation for many research areas, as the 3D color surface morphology of opaque samples—for example, intact adult *Drosophila*, *Xenopus* embryos, and other non-transparent samples—cannot be assessed. We have developed “ALMOST,” a novel optical method for 3D surface imaging of reflective opaque objects utilizing an optical projection tomography device in combination with oblique illumination and optical filters.

**Results:**

As well as demonstrating image formation, we provide background information and explain the reconstruction—and consequent rendering—using a standard filtered back projection algorithm and 3D software. We expanded our approach to fluorescence and multi-channel spectral imaging, validating our results with micro-computed tomography. Different biological and inorganic test samples were used to highlight the versatility of our approach. To further demonstrate the applicability of ALMOST, we explored the muscle-induced form change of the *Drosophila* larva, imaged adult *Drosophila*, dynamically visualized the closure of neural folds during neurulation of live *Xenopus* embryos, and showed the complementarity of our approach by comparison with transmitted light and fluorescence OPT imaging of a *Xenopus* tadpole.

**Conclusion:**

Thus, our new modality for spectral/color, macro/mesoscopic 3D imaging can be applied to a variety of model organisms and enables the longitudinal surface dynamics during development to be revealed.

**Electronic supplementary material:**

The online version of this article (10.1186/s12915-018-0614-4) contains supplementary material, which is available to authorized users.

## Background

Recent developments in 3D microscopy have revolutionized the imaging of whole model organisms such as zebrafish and *Drosophila* larvae as well as cleared mouse embryos and organs [1–3]. Among the techniques that allowed this revolution are light sheet or selective plane illumination microscopy (SPIM) [4, 5] and optical projection tomography (OPT) [6].

The 3D modality of these methods differ, with light sheet microscopy directly generating a stack of confocal sections and OPT acquiring multiple images collected at different angles by rotating the sample with respect to the image acquisition and back calculating the 3D information. This imaging mode bears similarity with micro-computed tomography (CT) and also means that standard tools for 3D reconstruction of CT data also work for OPT data.

Both light sheet and OPT devices require transparent samples to image fluorescence within the tissue. In addition to fluorescence, OPT has been used with transmitted light and absorbing dyes [6, 7], where the sample is back-illuminated and the light is partially absorbed as it passes through the transparent material. Different to light sheet, OPT is compatible with samples that slightly scatter light [8], but not suited for opaque samples. Therefore, apart from inherently transparent biological samples, like the zebrafish (*Danio rerio*), transparency needs to be induced using chemical “clearing” methods. These clearing methods are time intensive and are not compatible with all samples. Also, for studying the surface morphology and color appearance, optically clearing is counterproductive.

Therefore, for imaging the morphological details of opaque samples like adult *Drosophila* and the surface features of embryos, often brightfield dissecting microscopes are used that provide only a 2D representation of the 3D sample. Furthermore, similar scopes are also used to create extended focus operations [9], where a 3D stack is processed to provide a 2D projection of the in-focus parts of the stack, thereby losing the 3D information of the sample.

For 3D imaging of non-transparent objects, micro-CT is the gold standard and is based on the absorption of X-rays as they pass through the material [10]. X-rays interact differentially with matter compared to visual-spectrum photons and pass readily through biological material, which is why optically non-transparent samples can be imaged using a micro-CT. As the absorption of X-rays is visualized with a CT, both internal and superficial structures can be imaged at great detail. However, micro-CT is problematic for following morphological changes over time due to accumulation of radiation dose, which, for example, may influence the development during time-lapse experiments. It is also inconvenient for forward genetic screens searching for mutants with abnormal external morphology and related tasks, for two reasons. On the one hand, acquiring micro-CT devices poses a high financial burden for many labs and the interactive sample handling for optical methods is easier and potentially faster, meaning that more samples can be screened faster at a cheaper price with optical imaging than with micro-CT. On the other hand, micro-CT solely captures density differences of a sample and cannot detect optical properties like color appearance or differences in reflectivity. However, these optical properties (color, reflectivity) are readily accessible through optical imaging methods. For opaque samples, different from CT, optical methods can only depict the surface and cannot interrogate the inside of the object. Nevertheless, for many morphological questions such as imaging insect cuticles, mainly the surface of the sample is of high relevance.

### Rationale

Thus, there is currently an unmet need for straightforward 3D surface optical imaging of non-transparent biological samples, and such an approach would greatly benefit some fields and open novel possibilities for 3D quantitative biology.

Therefore, we created a new approach, complementary to the state of the art, aiming to visualize the optical properties of surfaces in 3D. We describe a new variation of OPT that provides a 3D surface reconstruction of opaque samples including information on color and reflective properties. We call our imaging approach A Label-free Multicolor (multi-wavelength) Optical Surface Tomography (ALMOST) method. It is based on the diffuse scattering of light that occurs when visible spectrum photons interact with the surface of nontransparent 3D objects. We show how this allows the 3D color visualization of a sample with a reflective surface. We test the applicability by reconstructing the 3D color surface from a diverse set of samples including an electrical resistor, seed cones of the dawn redwood *Metasequoia glyptostroboides*, the rosemary beetle *Chrysolina americana,* Lego figurines (which we compare with micro-CT), and a shell of the mollusk *Pollia dorbignyi* with six color channels. We also image the fruit fly *Drosophila melanogaster* (both larvae and adults) (including co-detecting GFP expression and genetic eye mutations) and image the surface of fixed and live *Xenopu*s embryos highlighting the applicability of our approach to detect shape changes, for example, during developmental furrow formations in neurulation. We predict that ALMOST will fill a gap for several research fields allowing documentation and quantitation on the mesoscale 3D surface color morphology of biological and non-biological samples.

## Results

**Fig. 1:**
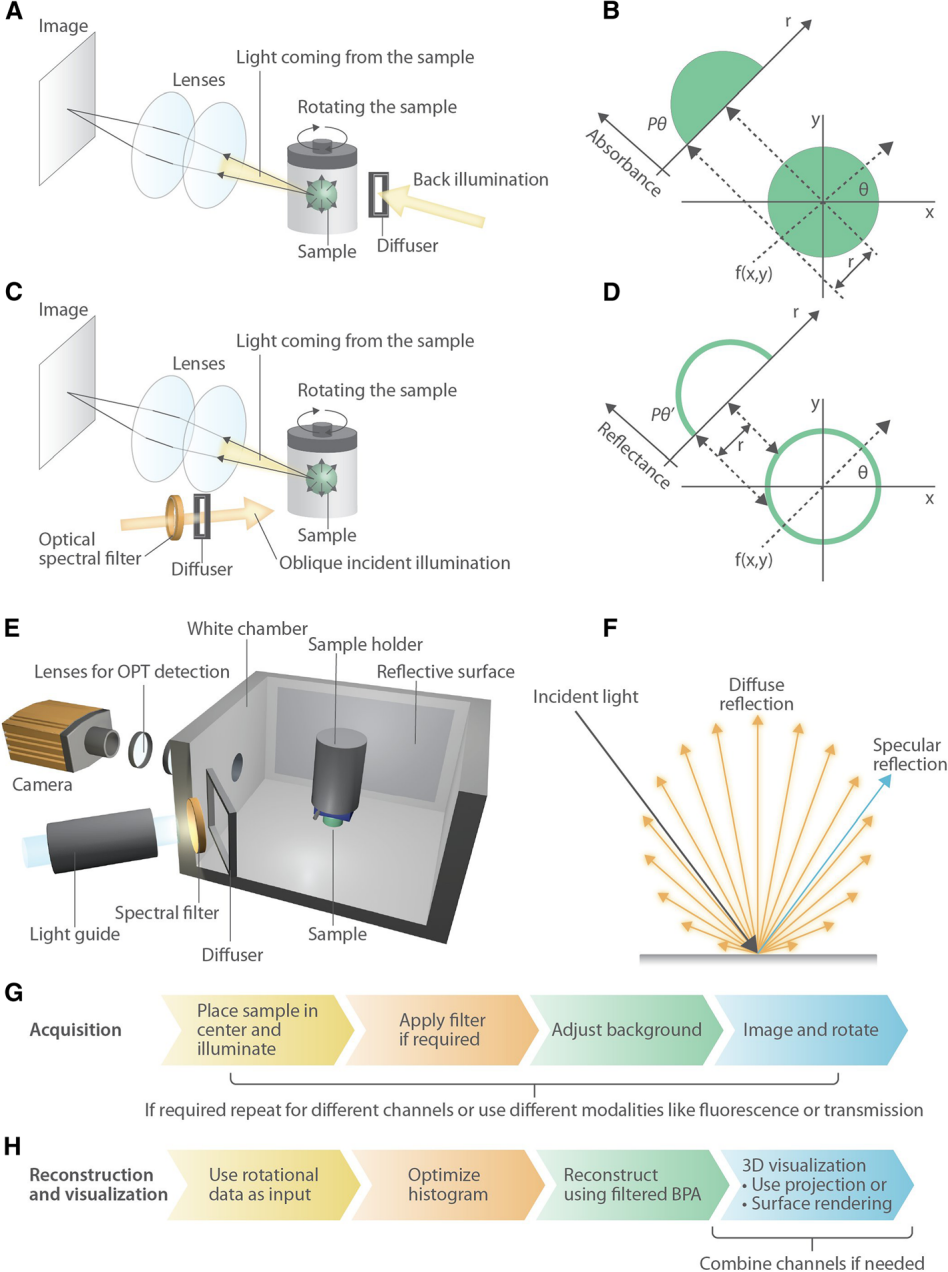
OPT and ALMOST imaging modalities. **a** Diagram of the imaging light path for a sample (green orb) with back illumination. **b** Theory of image formation in a tomographic system like OPT. The sample object resides at the center of a coordinate system. Parallel beams spaced by *r* pass through the sample to form a projected image (*P*_*θ*_). The theory of this imaging process is based on the Radon transform (see also Additional file 1: Text S1 for more detailed background; *r* is used in the CT imaging and is less relevant for light-based approaches). **c** Diagram of the oblique illumination light path to create reflected light images of opaque samples, while standard OPT works with transparent samples and uses fluorescence or back illumination. It is also possible to add color filters in the reflected light path to collect spectral information. **d** Theory of image formation when reflected light interacts with an opaque sample that contains surface topography information. **e** The oblique illumination/imaging chamber for reflected light imaging is depicted. It is crucial to use a reflective chamber, for example, lined with white paper, to promote diffuse illumination. **f** Depiction of diffuse reflection compared to specular reflection. **g**, **h** Flowcharts of the imaging, reconstruction, and visualization process. The filtered back projection algorithm is abbreviated as filtered BPA. It is of note that if NRecon is used for reconstruction that the images are converted and need to be inverted back (see Additional file 1: Text S1 for more information)

The two commonly used optical mesoscale imaging modalities are OPT and light sheet microscopy. As we wanted to image the surface, we pursued the aim of 3D color imaging using reflected light, given that opaque objects will absorb back-illuminated light and prevent a detailed image construction. As light sheet microscopy uses a perpendicular illumination scheme with respect to the light detection, we presumed it would not be the best option for reflective light detection. Therefore, we explored whether we can adapt an OPT device (Fig. 1) for 3D imaging of opaque samples.

For the reflective imaging, we developed an imaging chamber (Fig. 1e) that would promote diffuse illumination of an opaque object using commonly available materials. It contains a reflective background via white paper and aluminum foil, a non-coherent unfocused light source of LED goosenecks (like the ones used for dissection stereo-microscopes) directed at the sample, and a diffuser made of milk glass placed between the illuminator and the sample (Fig. 1c, e).

In this setup, light recorded by the detector includes rays that have reflected from the sample surface against a constant illumination background, meaning that the sample is imaged such that the absorption of light on its surface creates an image that is less bright than the white background, while no (or minimal) light interacts with the interior of an opaque sample (Fig. 1d).

Furthermore, the sample will differentially absorb and reflect light depending on surface properties like color, and thus, the reflected light image will contain spectral information about the sample.

The goal of the imaging chamber is to obtain images of the sample as if it were a self-radiant object. This is important, as this allows that the images mimic an absorbing or fluorescent sample and can thus be analyzed in a similar way. Therefore, we were aiming for diffuse reflection as compared to specular reflection (Fig. 1f). In diffuse reflection, the radiant or luminous intensity of a diffuse radiator is directly proportional to the cosine between the illumination direction and the surface (Lambert’s cosine law) [11]. That means that the surface reflection of light will scatter in different directions with the brightest reflection being perpendicular to the surface. When light is illuminating a sample from an indirect diffuse source, rays that are reflected at its surface can be captured by the objective lens of the OPT/ALMOST device and form an image on the detector (see Fig. 1d). This is different to standard OPT, where reflections are typically avoided by using immersion medium to match the refractive index of the sample. In contrast, when imaging in reflective mode, a refractive index mismatch is actually supporting the imaging. Thus, we imaged in air to maximize the reflectivity of the surface, except for “aquatic” samples like *Xenopus* embryos.

While diffuse reflected illumination may still lead to shadowing as in scanning electron microscopy (SEM), we reasoned that when a sample is imaged over a range of angles, as in OPT 3D imaging, the surface will be evenly illuminated and the topology can be imaged correctly. Furthermore, we hypothesized that multi-directional and even illumination of the sample would promote accurate detection of samples with complex and/or angular (non-convex) morphology while also gathering information on fine surface topography like dimpling.

After imaging and reconstructing the 3D information with the back projection algorithm, the next step is rendering and visualizing the imaged 3D shapes using 3D rendering software. In the context of transmitted light OPT and using standard processing of the back projection algorithm, darker parts of the image are considered as sites where rays are absorbed, while brighter parts are regions where rays pass unimpeded through the sample. In our case, brighter regions in reflected imaging are those where the sample has higher reflective properties. ALMOST imaging works with standard reconstruction software designed for absorption (NRecon). Thus, the images for ALMOST are inversed, according to the process introduced by the NRecon software (see Additional file 1: Text S1 for more information).

To render shapes in 3D acquired with reflective light imaging, it is essential that the illumination system provides a difference between the background intensity of light and the reflected light that has interacted with the sample. In addition, for 3D visualization, the background will typically be rendered transparent to reveal the 3D shape of the sample by a process called ray tracing. Our approach of using a white background achieves this for any non-white (or less bright) samples. Rendering the 3D shape of the sample also means that the result of ALMOST is a computer-generated object. This object can then be differently visualized as a projection, volume, or surface, where color information can be added in the form of a look-up table and illumination and shading can be animated. It also means that the result is a computer-generated image and thus may appear artificial as compared to a photograph. At the same time, it means that the 3D information is fully digitized and can be used for modeling printing, etc. The steps from imaging over reconstruction to rendering are illustrated in the flow diagrams in Fig. 1g, h.

We first tested in silico whether the back projection-based approach can reconstruct a 2D object from a series of images using MATLAB. The reconstruction of the simulated reflection from the outer surface of the phantom (Additional file 2: Figure S1) is showing the successful application of our approach applied to simulated reflected light images.

**Fig. 2:**
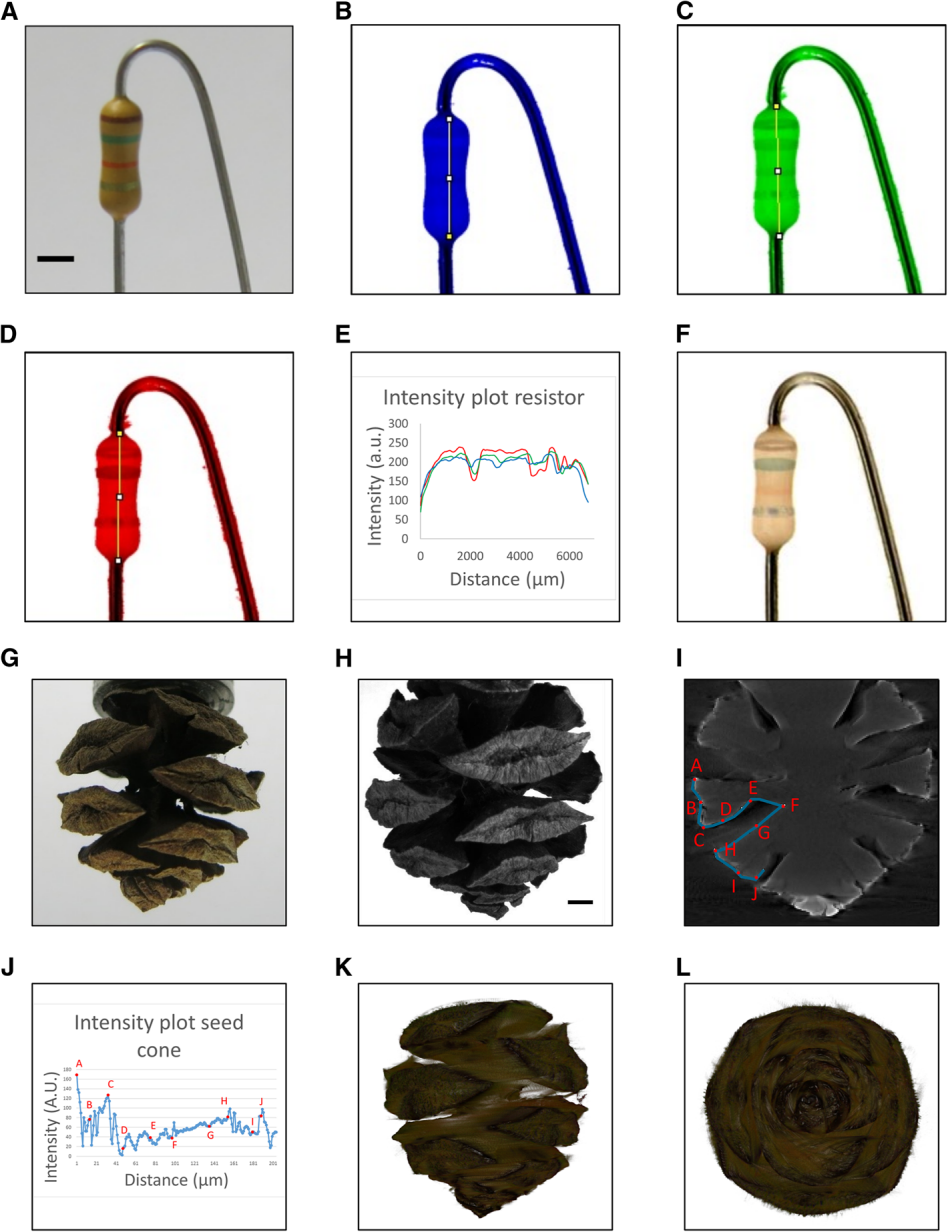
Color and shape in test samples. Color code on a resistor. **a** Photograph of the resistor. **b** 3D reconstruction of the blue channel. **c** 3D reconstruction of the green channel. **d** 3D reconstruction of the red channel. **e** Relative intensity profile of the color channels as indicated in **b**–**d**. **f** 3D RGB color reconstruction. Small artifacts like the flare on the thread above the actual resistor are a consequence of extended reflexes of the reflective metal part connecting the resistor. The brown, green, red, and gold color rings (from top to bottom) imprinted on the resistor, part of the four color code used to describe its properties, can be revealed. The color balance for the three colors was adapted manually. Seed cone sample (*Metasequoia glyptostroboides*) with complex surface structure. **g** Photograph of the seed cone. **h** Individual image from the ALMOST device (blue channel). **i** Reconstructed sagittal section through a central plane of the seed cone (blue channel). **j** Intensity profile along the line indicated in **i** to compare inside and outside of the complex shape. **k**, **l** 3D semitransparent volume rendering of the seed cone in three colors (colors inverted compared to **j**, for realistic color display). The color balance for the three colors was adapted manually. Images show different angles, including a view from below the cone, showing that complex samples can be imaged with ALMOST. Scale bars = 2 mm. Imaging conditions are summarized in Additional file 20: Table S1


We next tested the practical applicability of our approach to determine the true 3D shape of a sample with a relatively simple shape, but one that also includes color information. Pigmented specimens will differentially absorb and reflect different wavelengths of reflected light. The ALMOST device is equipped with a black and white camera, but we expected that we could generate color images of the sample using a set of three filters (Additional file 3: Figure S2) to create red, green, and blue color channels. The idea to use three color filters is akin to color photography as explored by James Maxwell in 1861 [12]. We used a resistor (Fig. 2a) to reveal the characteristic color code on its 3D surface in RGB color. Individual 512 × 512 pixel images were collected over 360° with 0.9° rotational steps. Reconstruction was based on the standard micro-CT NRecon software implementing the filtered back projection algorithm. The imaging, reconstruction, and rendering were carried out for the three RGB color channels (Fig. 2b–d). Figure 2e shows the intensity distribution along the lines indicated in Fig. 2b–d. We show a maximum intensity projection combining all colors in Arivis 3D rendering software (Fig. 2f and Additional file 4: Movie S1). In this projection, the sample appears partly see-through. Importantly, the color rings are revealed properly.

Additional file 4:**Movie S1.** Movie of the yellow resistor. (AVI 1042 kb)


To test if an automated procedure for adjusting the colors can be used and to show that the approach also works with a color camera, we imaged a resistor with a color camera and applied the automatic white balance (sometimes referred to as color balance) procedure from the software. The resistor was then reconstructed and visualized without individual adjustments for the different colors. Additional file 5: Figure S3 shows that the resistor can be depicted and the colors match the original well.

We next tested whether we can successfully reconstruct the complex shape of a biological sample. We chose a seed cone to test if reflected illumination can reveal a shape with cavities/non-convex morphologies. More specifically, we imaged seed-bearing cones of *Metasequoia glyptostroboides*, also called the dawn redwood tree (Fig. 2g; photograph). After imaging in the ALMOST device (Fig. 2h), we reconstructed the 3D structure, which allows to virtually slice through the seed cone showing its surface structure like it is cut open (Fig. 2i, Additional file 6: Figure S4). The intensity change of the signal for one of the cavities is shown in Fig. 2j. We again used red, green, and blue filters (Additional file 3: Figure S2) to create an RGB type of 3D image of the surface from three acquired volumes. Arivis 3D software visualized the surface of the seed cone in 3D (Fig. 2k, l, Additional file 7: Movie S2). Figure 2g–l and Additional file 6: Figure S4 demonstrate that ALMOST allows visualizing the cavities and complex structure of the object.


Additional file 7:**Movie S2.** Movie of the seed cone. (AVI 2365 kb)


The aim of our imaging chamber is to create diffuse illumination that should enable imaging of glossy samples. To test and confirm this, we imaged a *Chrysolina americana*, commonly known as the Rosemary beetle. These insects have a colorful elytra with metallic green and purple stripes along the rostral to caudal direction on them. Additional file 8: Figure S5 shows that ALMOST can image these smooth, shiny surfaces and visualize the color pattern and typical indentations on the forewing of the beetle. To also test inorganic metallic surfaces, we imaged a regular eurocent coin. The oak leaf imprint of the German mint becomes visible. Additional file 8: Figure S5 also demonstrates the applicability of ALMOST for imaging the 3D morphology of insects and metallic samples.

**Fig. 3:**
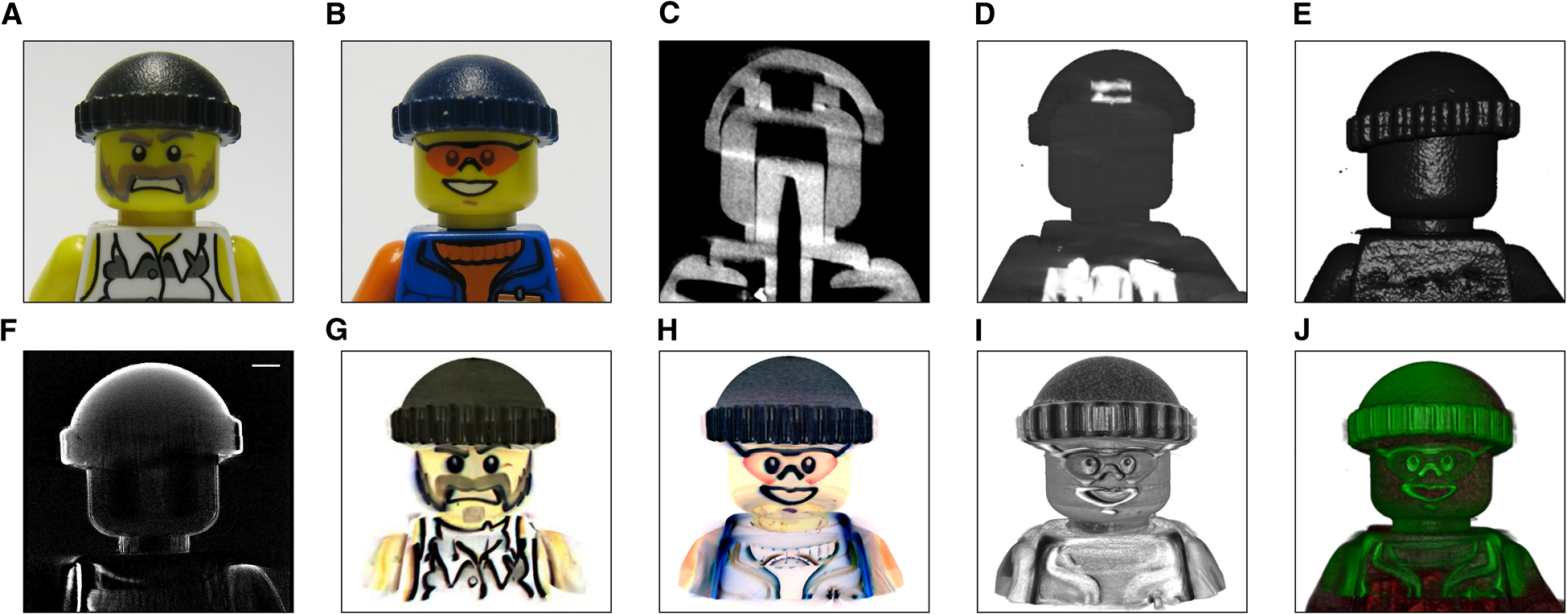
Micro-CT and ALMOST imaging of the same samples. Samples of the same shape but with different color patterning are imaged, namely Lego figurines. **a**, **b** Photograph of a Lego figurine with a beard dubbed “Dalton” and of a Lego figurine with glasses and happy face dubbed the “Workman.” **c** A reconstructed sagittal section from the Dalton obtained with micro-CT. **d** Dalton maximum intensity 3D reconstruction of the micro-CT data made similarly as the ALMOST data (with Arivis). **e** Dalton volumetric 3D reconstruction of the micro-CT data. **f** A reconstructed sagittal section from the Dalton obtained with ALMOST, blue channel. **g** Dalton maximum intensity 3D reconstruction of the ALMOST data, made with Arivis. **h** The Workman maximum intensity 3D reconstruction of the ALMOST data, made with Arivis. **i** Using ALMOST, the surface can be revealed similar to the depicted micro-CT surface (cfr **e**) using, for example, one color channel (here blue). **j** Overlay of the surfaces from ALMOST (green) and micro-CT imaging (red). The ALMOST surface matches well the CT surface, thereby showing that the retrieved surfaces can be quantitative. Both characters are revealed in ALMOST imaging and can be discriminated, whereas in micro-CT, the figurines look similar. Optical imaging conditions are summarized in Additional file 20: Table S1. Scale bar = 2 mm


To compare the standard for imaging optically nontransparent samples, micro-CT, with our ALMOST imaging method and stress the unique possibilities that the color imaging holds, we imaged samples that are similar with respect to their 3D shape and surface, but with different coloring. We used the reproducible shape of Lego figurines and imaged two figurines with different facial expression and color scheme (Fig. 3a, b). Using micro-CT, the shape and surface of the figurines can be depicted, but the two characters cannot be discriminated (Fig. 3c–e). Using ALMOST, the two characters can be clearly discriminated, also the surface of the figurines can be extracted using volume rendering similar to the CT data (Fig. 3f–i). The surface extracted from the ALMOST imaging and the CT surface match well showing qualitatively that the surfaces are comparable to CT surfaces of similar resolution (Fig. 3j, Additional file 9: Movie S3).

Additional file 9:**Movie S3.** Movie of the overlay between ALMOST and micro-CT data. (AVI 2199 kb)


To compare the performance of ALMOST with conventional OPT imaging and explore the resolution further, we used electron microscopy grids, beads, and *Drosophila melanogaster* eyes as test samples (Additional file 10: Figure S6). Overall, the reconstructions of both modalities match well, while a section through the grid for both modalities reveals potential artifacts for ALMOST in the form of specular reflections. The bead imaging revealed that the sampling of the camera is the limiting factor in the current setup.

Subsequently, we explored if we could use our approach to go beyond the three-color RGB images and get a more extended spectral readout from the sample surface. To this aim, we imaged a sea snail shell using six spectral filters. Additional file 11: Figure S7 and Additional file 12: Movie S4 show the shell of *Pollia dorbignyi* in 3D color and a plot of the spectral composition of different parts of the shell and plasticine used to hold the shell in place. The plot is based on six volumes acquired with six spectral filters. This demonstrates the applicability of ALMOST for imaging shells of mollusks and generating multi-color spectra.


Additional file 12:**Movie S4.** Movie of the shell. (AVI 1578 kb)


Recent studies using light sheet microscopy created impressive sequences of the early *Drosophila* development using fluorescent nuclear markers showing the inner cellular organization of the larvae. These movies end when the muscles of the larva start twitching [13]. To illustrate the complementarity of our label-free surface approach, we demonstrate as a proof of concept the morphic potential meaning the amount of form change in a *Drosophila* third instar larva. The movements of the *Drosophila* larva, for example, during foraging, are characterized by a remarkable form change of the larval body. We reasoned that a tomographic view provides more insights than the obvious macroscopic contractions. We show a larva in a contracted, curled-up, and relaxed elongated state. This reveals that the contractibility and deformability of the larval body differs along the different axes (Additional file 13: Figure S8). Hence, these results show that the morphic potential of this individual is manifested unequally between the different axes and is pointing to a differential contribution of the different muscle groups involved in the movement.

**Fig. 4:**
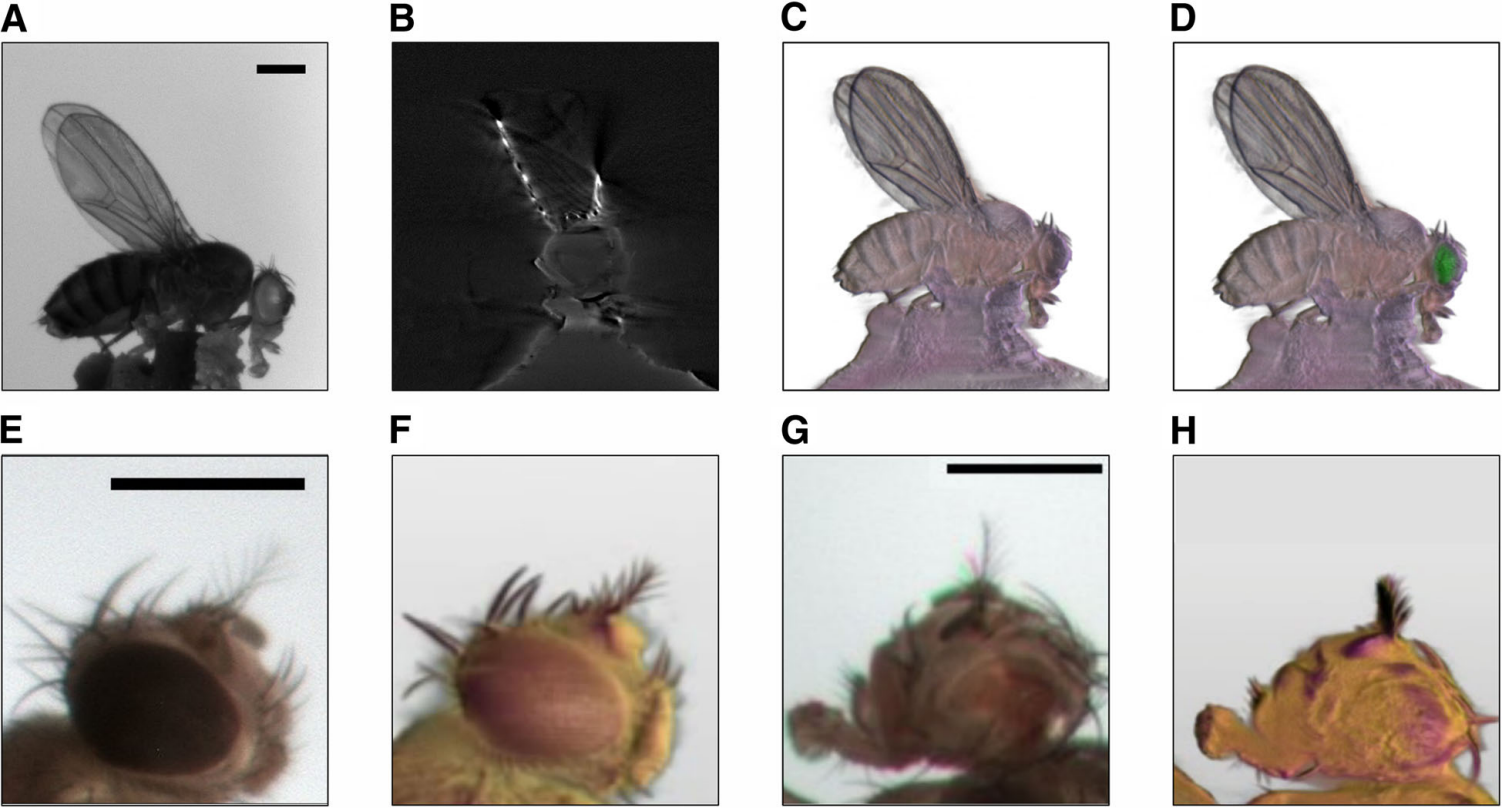
Combining ALMOST and fluorescence OPT on adult *Drosophila.* A mutant fruit fly expressing GFP in the eyes is imaged. **a** shows the reflective image acquired by ALMOST in the blue channel. **b** Section through reconstruction of the fly in reflective mode (front view, blue channel). **c** 3D rendering of the fly imaged in reflective mode. **d** Combination of the 3D rendering of the reflective and the fluorescence modes. **e** Single three-color (raw) ALMOST image before reconstruction and rendering of a red-eyed wild-type *Drosophila* head. **f** ALMOST 3D reconstruction of a sequence of rotational images as in **e**. **g** Single three-color ALMOST image (before reconstruction and rendering) of a glace-eyed mutant *Drosophila* head with narrowed eyes of reduced size. **h** ALMOST 3D reconstruction of a sequence of rotational images as in **g**. Scale bars = 500 μm. Imaging conditions are summarized in Additional file 20: Table S1


Next, we tested if ALMOST can be used for imaging adult *Drosophila melanogaster* fruit flies. We tested the combination of ALMOST imaging with fluorescence using a mutant fly expressing GFP in the eyes. In Fig. 4a–d and Additional file 14: Movie S5, we highlight that ALMOST can be combined with fluorescent OPT. We can reconstruct the overall surface morphology of the cuticle of the fly in 3D and add an additional channel for fluorescence detection.

Additional file 14:**Movie S5.** Movie of the fly with fluorescent eyes. (AVI 1699 kb)


To further test the applicability for imaging fly mutants and to test the resolution of our setup on biological samples, we imaged the heads of wild-type red-eyed *Drosophilas* (Fig. 4e, f) and so-called glazed eye (Gla) mutants with narrowed eyes of reduced size (Fig. 4g, h).

Using ALMOST, the eye morphology of the fly can be imaged in 3D, and the difference between the two genotypes can be detected (see also Additional file 15: Movie S6 and Additional file 16: Movie S7). Figure 4 underlines the applicability of our method for imaging the morphology of insects.


Additional file 15:**Movie S6.** Movie of the eye morphology of a wild-type fly. (AVI 953 kb)



Additional file 16:**Movie S7.** Movie of the eye morphology of a mutant fly. (AVI 1484 kb)


Next, we wanted to test if we can image the embryogenesis in *Xenopus* embryos. *Xenopus* is a commonly used model system and widely used for embryology studies. *Xenopus* eggs and embryos are opaque, likely because of their yolk content, which is different to some other model organisms like zebrafish and *Drosophila* embryos that are more transparent. Recently an adaptive light sheet microscope was introduced to overcome spatially varying optical properties in tissue and to image embryo development in greater detail. This technique allows improving live cell imaging in *Drosophila* and zebrafish embryos [14]. However, such a system can only correct for varying cell density. In opaque and absorbing systems, where the signal is lost, such as, for example, in *Xenopus* embryos, it is of limited use. To overcome the opacity issue, recently, microscopic magnetic resonance imaging (mMRI) was used to investigate noninvasively and independent of the optical properties mitotic divisions inside the opaque early *Xenopus* embryo [15] and has been successfully applied to unravel disheveled signaling in *Xenopus* gastrulation [16]. Other solutions for imaging opaque and highly scattering samples have been proposed before like surface imaging microscopy for example [17], which has been used to investigate embryo development of fixed samples. To test if we can image *Xenopus* embryos with ALMOST, we looked at chemically fixed embryos at different stages. Additional file 17: Figure S9 shows examples of embryos at the one-cell stage (stage 1), four-cell stage (stage 3), blastula stage (stage 7), large yolk plug stage (stage 11), neural plate stage (stage 14), mid neural fold stage (stage 15), early tailbud stage (stage 25), and a tailbud stage (stage 28). This shows that with ALMOST, we can image the surface of *Xenopus* embryos label-free and without clearing.

To test, if we can leave out all chemical treatment altering the sample (clearing and fixation) and thus image live *Xenopus* embryos, we next looked at the process of neurulation during *Xenopus tropicalis* development. We are interested in the neural tube formation and especially in the late steps from fold apposition to fusion and remodeling [18]. This process is of relevance as the failure to close the neural tube can lead to neural tube defects. Neural tube defects (NTDs) are one of the most common birth defects affecting approximately 0.5–2/1000 pregnancies [19]. These NTDs include spina bifida and anencephaly [20]. *Xenopus* is a good model system for spinal cord formation [21, 22], as the vertebrate-specific program of neurulation can be observed easily outside the uterus. While zebrafish would potentially be an alternative and inherently transparent model system that can be imaged with the available techniques (light sheet microscopy), the process of neurulation differs, and the zebrafish undergoes so-called secondary neurulation [23], which is different from the more human-relevant primary neurulation. Therefore, being able to image and analyze the neurulation in alive *Xenopus* embryos is an advantage.

**Fig. 5:**
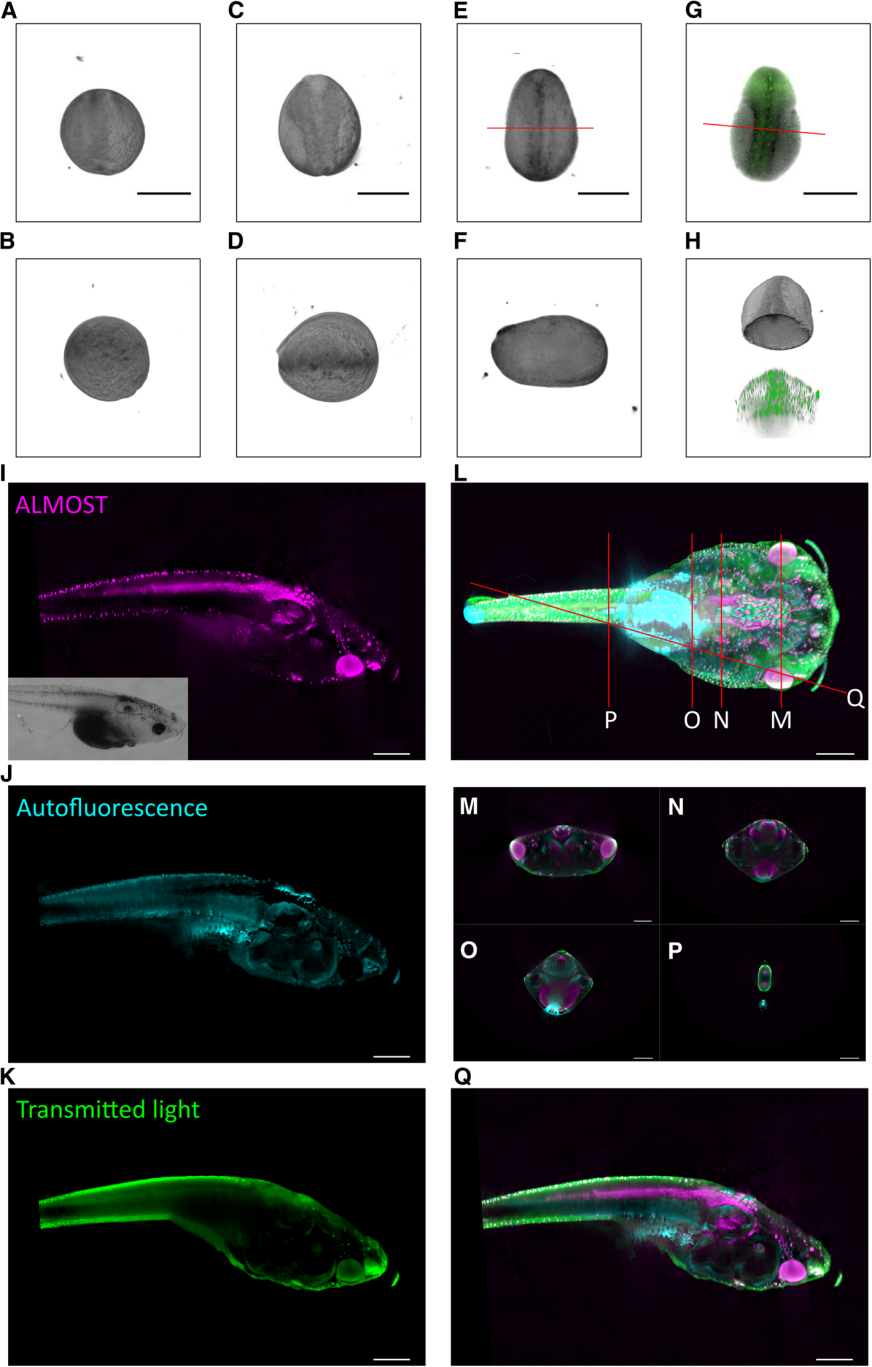
Live imaging of a *Xenopus tropicalis* embryo; complementarity of the ALMOST approach. Different stages of the same *Xenopus tropicalis* embryo are shown during neurulation. **a** Top view from dorsal to ventral side of a stage 12 embryo. **b** Lateral side view of **a**. **c** Same embryo as in **a** and **b** after ~ 1.5 h. Stage 14.5 is shown. **d** Lateral side view of the embryo in **c**. **e** Same embryo as in **a**–**d** after ~ 2.8 h (relative to **a**, **b**). Stage 19 is shown. **f** Lateral side view of **e**. **g** The GFP fluorescence signal of the same embryo is shown after fixation and imaged with a spinning disk. Gray information is the transmitted light signal from the spinning disk. The embryo is a crest3- gfp transgenic line, labeling a subset of neuronal precursor cells. **h** Zoomed view, comparing **e** and **g**. Horizontal section view of the embryo from anterior to posterior and cut open at the line indicated in **e** and **g**. As the animals get older, they become more transparent. To demonstrate the complementarity of our approach, we imaged a semitransparent tadpole of stage ~ 50 with ALMOST, with fluorescence OPT, and with transmitted light OPT. **i** Side view using ALMOST displayed in purple, with more brightness in purple indicating less reflection. Insert is showing a raw reflection image. **j** Side view using autofluorescence displayed in cyan. Brighter signals indicate stronger autofluorescence. **k** Side view using transmitted light displayed in green. Brighter signals indicate lower transmission. **l** Merged view showing the tadpole from the top. **m**–**p** Virtual sections through the animal as indicated in **l**. **q** Merged side view of **i**–**k**, section as indicated in **l**. The different approaches reveal different aspects of the tadpole. Part of the difference between transmitted light and ALMOST is due to scattering inside the sample. Scale bar = 500 μm in all images. Imaging conditions are summarized in Additional file 20: Table S1


For studying the neural tube closure, surface imaging is crucial as exemplified by earlier SEM studies [18]. Using ALMOST, we imaged the furrow and the progress of the closure of the neuronal tube between stages 12 and 19 during a period of ~ 2.8 h in four steps. Figure 5a–f (and Additional file 18: Movie S8) shows that with ALMOST, developmental processes can be studied in 3D and the surface dynamic of live embryos can be imaged. As this embryo expresses GFP in neuronal precursor cells, the neuronal tube formation can also be studied in this respect. To demonstrate how ALMOST complements other imaging strategies and to see how it compares to already available 3D imaging strategies, we imaged the same embryo after chemical fixation on a spinning disk microscope. Figure 5g shows the widefield and fluorescent signal obtained from the spinning disk microscope. Figure 5h highlights a zoomed 3D view, where the embryo was cut digitally, and the furrow is shown towards the caudal direction. The signals from the spinning disk and ALMOST are shown next to each other. One can see how the fluorescence provides specificity for identifying the precursor cells and how ALMOST provides the context visualization of the reorganization of the tissue. Figure 5a–h signifies that opaque model systems can be imaged using ALMOST.

Additional file 18:**Movie S8.** Movie of *Xenopus* stage 19. (AVI 577 kb)


As the *Xenopus* gets older, it gets more transparent. Therefore, we wanted to check next if we can image semitransparent samples and compare our technique with other related OPT techniques. As proof of concept, we image a technical semitransparent sample, a LED, to show that with ALMOST, internal structures can be revealed (Additional file 19: Figure S10). Next, we have imaged a tadpole of stage ~ 50 with ALMOST, autofluorescence, and transmitted light OPT. Figure 5i–q shows that semitransparent samples can be imaged with ALMOST; it also shows that the information it reveals is different from the transmitted light and the autofluorescence signal underlining the complementary character of our approach.

## Discussion

Here, we introduce the characteristics and applications of a novel (mesoscopic) optical imaging technique. We call this approach A Label-free Multicolor Optical Surface Tomography (ALMOST) method. We have shown that we can image opaque samples in 3D and that the shape can be revealed in color by combining OPT with oblique illumination and color filters and using the filtered back projection algorithm together with 3D rendering software. This approach overcomes the need for transparent or cleared samples and allows the analysis of the 3D morphology of opaque samples like insect cuticles or shells on the mesoscale. As non-fixed and non-cleared samples can be imaged, ALMOST opens the possibility for longitudinal imaging of unaltered, live samples. As it reveals complementary information to transmission and fluorescence, it poses an ideal supplementary approach to well-established OPT and light sheet modalities and allows imaging of the sample color, which is lost in X-ray-based techniques like micro-CT.

We applied ALMOST to the surface of samples like seed cones, adult insects, resistors, and Lego figurines using straightforward modifications of existing OPT hardware, underlining that complex (Fig. 2) and multicolor (spectral) samples (Additional file 11: Figure S7) can be imaged with ALMOST and that reconstructed surfaces are in accordance with CT imaging (Fig. 3).

Next, we demonstrated that ALMOST opens the possibility to quantify morphological differences in 3D (Figs. 4 and 5) and could be used for quantifying genetic interaction screens in model organisms. Genetic interaction studies in *Drosophila* often use eye deformations as readout. However, as compared to the 2D extended focus operations [9] that are the standard for *Drosophila* eye imaging or SEM, which is tedious, ALMOST allows imaging and quantifying the morphology of the eye (Fig. 4) and other morphological features (Additional file 13: Figure S8) straightforwardly in 3D.

The *Xenopus* imaging shows that ALMOST can be used to image live samples and opens the possibility for longitudinal non-destructive surface imaging of the developmental process. It also highlights the potential of ALMOST for *Xenopus* embryogenesis (Additional file 17: Figure S9) and for investigating critical steps of neurulation (Fig. 5). The commonness of neural tube defects during pregnancies [19] stresses the importance of this topic. The fact that *Xenopus* has recently become of interest for high-content screening [24] further supports the relevance of this proof of concept, especially as ALMOST could as well be integrated into robotic workflows. For the quantitative measurements of complex features on the embryo, a coordinate system like it has been developed for spherical embryos [25] or other frameworks for modeling embryogenesis [26] could be applied.

The possibility to use reflected visible light with low intensities compared to fluorescence means as well that the sample is treated gently and that phototoxic effects are reduced.

The combination of our technique with fluorescence OPT (Fig. 4) and the images from the semitransparent tadpole sample (Fig. 5) show that our approach is easily combinable with other imaging modalities and can add to the already available repertoire of imaging techniques. The fact that we used a modified OPT device opens new applications for this straightforward imaging technique. Due to the difference in the imaging geometry, these applications are not directly available in light sheet imaging, where shadowing will play a more prominent role. However, a similar addition of a diffuse light source and rotation of the sample, sometimes already implemented in light sheet approaches, opens the possibility for a combined light sheet/ALMOST imaging device [27–29].

The achievable resolution is given by the optical system. Different methods for characterizing the resolution are being used [30] including the Abbe diffraction limit [31], which would be given by the wavelength used divided by two times the NA of the objective lens. In OPT imaging, the 3D reconstruction can approximate isotropic resolution for an increasing number of angles used in the reconstruction. However, in ALMOST, artifacts stemming from specular reflection may influence the images (Additional file 8: Figure S5). Thus, key for the reconstruction is to avoid highlights or specular reflections and image diffuse reflection. Additional file 8: Figure S5 shows that glossy surfaces can be imaged with ALMOST.

For OPT and related types of imaging, like ALMOST, a practical limitation is the tradeoff between depth of focus and resolution, to prevent the images from being affected by out-of-focus blur. Practically, this means that the availability of objective lenses and sample geometry/size dictate the achievable resolution. The use of point-spread-function-aware algorithms for reconstruction [32] can improve the achievable resolution and reduce out-of-focus information, allowing higher NA lenses to be used. For an overview of used voxel sizes, please refer to Additional file 20: Table S1. It is of note that the different wavelengths in the multicolor images will have a different resolution with longer wavelengths being more strongly diffracted.

Other schemes to retrieve the spectral information are possible, including the use of a monochromator, color cameras (Additional file 5: Figure S3), different spectral light sources such as colored LEDs, and a second camera combined with a dispersive element to access pixel-wise spectra. Similar to other color and spectral imaging techniques, problems with balancing different color channels can arise and normalization of the different stacks may be required (Additional file 5: Figure S3 and Additional file 11: Figure S7). Aligning different color stacks due to mechanical shifts between repeated imaging for multi-color retrieval may be required and needs to be taken into account, for instance, by manual alignment using landmarks either on the reconstructed images or in the 3D visualization software akin to the OPT to CT alignment in Fig. 3. For a more sophisticated way of combining colors than performed here, a phasor-based system like it that was described earlier may be applied [33].

The use of an RGB camera for imaging is a straightforward way to speed up the imaging process for multicolor imaging and reduce the number of separate channels to be recorded in ALMOST. The acquisition of a 360° view of the sample on our device with a voxel size of 37.91 in x,y,z typically was in the range of 3 min/channel. The use of a faster rotational stage that is directly triggering the camera can speed this up (see Additional file 5: Figure S3). The processing on a standard computer workstation (Dell Precision 3500) from 2010 with the filtered back projection algorithm was in a similar range.

The 3D reconstruction used is based on the filtered back projection algorithm typically used in CT (Additional file 2: Figure S1). The fact that it can be used for reflective surfaces poses a new application for that reconstruction as it is originally based on the idea of line integrals typically associated with the attenuation of rays traversing through an object. Adaptations for reflective imaging, like filtering for small specular artifacts, as in Additional file 10: Figure S6, could be beneficial for future developments. The use of a telecentric lens might be improving the reconstruction additionally because of the reduced perspective skewing in OPT and ALMOST.

Due to the rotation of the sample, the surface of an opaque sample imaged by ALMOST can be more complete as compared to the same surface imaged by a confocal microscope [34]. As a result of the imaging geometry of the confocal, where the sample is illuminated and imaged through the lens, the backside of an opaque sample can be concealed due to the opaque nature of the sample. However, shadowing or shielding by nested surfaces can impact the reconstruction in ALMOST imaging, and as concavities will be visible under fewer angles, the reconstruction quality may suffer. Nevertheless, a complete 360° view of non-convex samples can be recovered by ALMOST imaging (Fig. 2). For opaque samples, no information from the inside is retrieved by reflective imaging. However, from Additional file 2: Figure S1, Additional file 19: Figure S10, and Fig. 5, it can be deduced that principally semitransparent and nested surfaces can be reconstructed given that the reflectivity of inner layers is higher or of a different wavelength than outer layers and the dynamic range of the system allows them to be discriminated. This opens the possibility to image internal surfaces with a different refractive index, e.g., swim bladders in fish or compound samples where a surface of interest is wrapped into a transparent conduit.

The ALMOST application is different from extended focus operations, where only a 2D projection is created from the acquired 3D stack. Nevertheless, ALMOST can benefit from similar ring illumination schemes and solutions to reduce highlights, known from material sciences applications [35], and macro photography6 [36].

ALMOST is novel as it differs from known technologies like optical coherence tomography (OCT) [37], as it is not utilizing an interferometric approach. It is as well different from earlier described optical reflection tomography [38] as it is not based on measuring the refractive index and the thickness of the sample. In contrast to our method, diffuse optical reflection microscopy utilizes a single continuous wave laser for illumination of the sample [39]. High-resolution reflection tomographic diffractive microscopy has been proposed earlier. However, a holographic approach and high NA lenses for imaging were used [40] instead of an OPT to target the mesoscale. It is also different from the recently published reflective light sheet microscopy, which aims to use reflective surfaces to increase fluorescent signals [41], or optically sectioned imaging by oblique plane microscopy [42]. The imaging of reflective samples in this application is different from the fluorescence OPT imaging described by Sharpe and colleagues [6], as here different properties of the material are probed. Nevertheless, as the reflective imaging is nondestructive, it can be combined with fluorescent OPT imaging (Figs. 4 and 5). However, for nontransparent samples, only fluorescence from the surface can be retrieved.

For scanning 3D objects on the macro scale, several methods are known but differ from ALMOST, like photogrammetry [43] which uses information like the focal length of the lens for making its calculations. Likewise, ALMOST is different from triangulation-based laser scanners; structure reconstruction from motion, modulated, or structured light scanners; and point cloud systems scanners [44, 45], as ALMOST uses a continuous angle acquisition and algorithms from CT processing. ALMOST imaging is contact-free; it is also different from methods combining silhouettes as intensity information is collected. It is not working by the time of flight [46] or requires a conoscopic crystal like in conoscopic holography [47]. ALMOST differs from 3D light scanning macrography, which is having a similar goal, but moves the sample through sheets of light linearly, where shadowing can have bigger effects [48]. Interestingly, in a dissimilar approach, surfaces have also been retrieved earlier, by interpreting the shadow cast by the specific topology of an object and removing it from an estimate of the object [49], with the estimated being generated by images of silhouettes.

Finally, no user-assisted image-based modeling is required with the use of computer-aided design (CAD) or related programs. As such, the ALMOST principle is not limited to the micro/mesoscale and is also applicable to larger samples as this method is non-invasive and could be expanded to “3D-spectral-virtual photography” including applications for recording biometry data and 3D representation of goods.

A comparison with most commonly used 3D imaging tools can be found in Table 1.

**Table 1:** Comparing ALMOST with other common 3D imaging techniques. + and − signs are indicating how well the different techniques are suited for the challenges in the category listed in the columns on the left side

	ALMOST	CT	Light Sheet	OPT	Confocal
Labeling					
Label-free imaging	++	++	−(+)	+	+
Fluorescence	+	−	++	+	++
Multiplexing	+	−	+	+	+
Data					
Isotropic	++	++	+	++	−
3D	++	++	++	++	+
Color	+	−	−	+	+
Samples					
Live	++	+	++	++	+
Mesoscale	+	++	+	+	−
Microscale	−	+	+	−	++
Opaque	++	++	−	−	−(+)
(Semi-) transparent	++	++	+	+	+
Scattering	+	++	−	−	−

## Conclusion and perspectives

We believe that ALMOST imaging will be beneficial to study and document non-invasively and non-destructive the 3D morphology of samples, where the surface is of interest such as insect cuticle, plant seeds, alive and developing *Xenopus* embryos, and mollusk shells. ALMOST fills a gap for cost-effective and accurate 3D surface characterization. The ability to record the surface of a mesoscale object in 3D opens perspectives for digital repositories of zoological and botanical collections and enables a link to 3D printing of these objects. In addition, the possibility for spectral analysis can provide more insight into the pigments in the samples and may allow applications for diagnostics of small parts in material science, like, for example, the amount of oxidation and point of failure analysis in industrial processes. Other applications may include virtual reality and numerical simulations of 3D objects, but also art and historic objects, including the analysis of coloring on ancient statuary and pottery and the teaching of these.

Our technique complements other valuable approaches, such as OPT, micro-CT [50, 51], X-ray microscopes, or light sheet microscopy, for 3D representation of the sample’s surface morphology thereby adding complete preservation of the actual characteristic color scheme without the need to use contrast agents, sample preprocessing, or digital post-processing to reintroduce the colors. ALMOST will not reveal the inside of opaque samples, but it is cheaper and safer than a micro-CT, can be implemented straightforwardly, and is well suited for field applications. Our approach is compatible with recently described resources for cheap custom-build OPTs [7]. Also for 3D rendering, open solutions like Drishti can be used [52]. Given the fact that the device is cheap and online resources are available for building OPTs, we expect quick acceptance and implementation of this novel imaging application.

## Methods

### ALMOST device

We used a SkyScan 3001 M OPT scanner, manufactured at Bruker micro-CT, Kontich, Belgium, for BIOPTONICS (Bioptonics, MRC Technology, Crewe Road South, Edinburgh, EH4 2SP, UK), with the following technical modifications:

#### Diffuse oblique illumination

To create an even diffuse illumination, we used a diffusor and lined the imaging chamber with white paper. The sample was illuminated from the side with a gooseneck LED (Leica KL 200 LED) white light source. Aluminum foil at the other side of the sample reflected light on the non-illuminated side.

#### Filters

A K580 from a Leitz filter slider was used as a red filter. As a green filter, a Leitz Gelbgrün 32-mm/35-mm color glass was used. As blue filter, a Leitz CB 16.5 blue filter with diameter 32 mm/35 mm was used (for spectral information, see Additional file 3: Figure S2). The bandpass filters used in Additional file 11: Figure S7 were a 377/50-nm filter provided by Zeiss, a 420/40-nm filter provided by Olympus, a 460/50-nm filter provided by Nikon, a 525/50-nm filter provided by Zeiss, a 600/50-nm filter provided by Olympus, and a 690/70-nm brightline filter provided by Semrock.

For Additional file 5: Figure S3, we used a DFC450c camera from Leica microsystems (Wetzlar, Germany) steered by Leica LAS software (version 4.8), attached to a Nikon (Tokyo, Japan) Te200 stand outfitted with a Nikon Plan Fluor 4× lens with 0.13 NA and 16.5 mm working distance. The sample was rotated using a Xeryon (Leuven, Belgium) XRT-U 30 rotational piezo stage.

#### Micro-computed tomography

Micro-CT datasets were acquired on a SkyScan 1278 (Bruker micro-CT, Kontich, Belgium) in step-and-shoot mode with the following parameters: 65 kVp X-ray source voltage and 770 μA source current combined with an X-ray filter of 1 mm aluminum, 40 ms exposure time per projection, four averages per view, acquiring projections with 0.7° increments over a total angle of 180°, resulting in reconstructed 3D datasets with 50 μm isotropic voxel size.

#### Image reconstruction

For ALMOST, OPT, and micro-CT reconstruction, NRecon 1.7.1.6 micro-CT software from Bruker was used.

#### 3D visualization

For the 3D visualization of the reconstructed stacks, we used Vision4D from Arivis 2.12.5 (Unterschleissheim, Germany). It is of note that for different 3D rendering software, slightly different ways to implement lightning and color exist. For more information on visualization and data treatment for visualization, please see Additional file 1: Text S1. For the instructions on the tools we used, we refer to the documentation part of Additional file 1: Text S1 and Additional file 21: Table S2 for the visualizations used in the manuscript.

For the imaging, we used a white background. That means that regions as bright as the background or brighter will be revealed as see-through. Thus, depending on the variation of brightness of the sample, the illumination needs to be adapted to low levels in order not to lose the bright regions in volume rendering. This can pose a limitation depending on the dynamic range of the camera and the possibility to illuminate the background as well.

The CT and ALMOST volumes were aligned using the ec-CLEM plugin from Icy [53]. Five landmarks (points) were added in order to compute the transformation in 3D.

#### Simulation

Simulations were performed using MATLAB 2016b (Mathworks, MA USA), the MATLAB toolbox DIP*image* 2.8 (TU Delft), and Fiji/ImageJ [54]. For Radon and inverse Radon transforms the “radon” and “iradon” commands from the image analysis toolbox were used. The simulation is using the Shepp-Logan head phantom [55].

#### Samples

The *Metasequoia* seed cone was collected at the Leuven botanical gardens.

The resistor is a 15-kΩ resistor with a tolerance of 5% and has the four band resistor codes: brown, green, red, and gold, purchased from R&S (RS Components GmbH Hessenring 13b, 64546 Mörfelden-Walldorf).

Figurines are from LEGO™ (Billund, Denmark).

The *Chrysolina americana* sample was collected approximately at 50° 85′ 82.49″ N, 47° 04′ 25.3″ E.

The *Drosophila* samples were fixed at − 80 °C to maintain the morphology and fluorescence. The fly strain used in Fig. 4a–d expresses GFP in the eyes in a white-eyed background (genotype: y[1] M{vas-int.Dm}ZH-2A w[*]; Bloomington stock center # 24481). Fly strains used in Fig. 4e–h were Canton-S (CS), Kyoto stock center # 105666, and w; GlaBC/CyO (Bloomington Drosophila stock center # 6662).

Grids were square mesh EM support grids, 400 copper mesh with 26 μm bars (FCF 400 – Cu – SB Electron Microscopy Science) and a finder grid with 17 μm bars (Agar scientific).

Beads were magnetic Dynabeads 500 with iron core with ~ 5 μm size (Thermo Fisher).

A *Pollia dorbignyi* [56] shell was used for the spectral imaging and was obtained at 42° 21′ 49.7″ N, 3° 09′ 47.2″ E.

Samples were mounted using plasticine.

For Additional file 13: Figure S8, a third instar larva was collected from the food of ongoing fly culture and washed in tap water. The wet larva was stuck to the insect pin by adhesion. For imaging, the larva was exposed to an atmosphere of CO_2_ to stop it from moving during the acquisition. A 0.2 M NaN_3_ solution for 30 min was used to relax the muscles [57].

*Xenopus tropicalis* embryos were placed in FEP tubes, with 1.6 mm diameter for imaging. Fixed embryos were imaged in PBS, while living ones were kept in 1/9th diluted Modified frog Ringer (MR: 0.1 M NaCl,1.8 mM KCl, 2.0 mM CaCl_2_, 1.0 MgCl_2_, 5.0 mM, HEPES-NaOH (pH 7.6), or 300 mg/l NaHCO_3_) solution. The tube with the frog embryo was submersed in a buffer containing glass cuvette during acquisition. For the live imaging, a crest3-gfp transgenic reporter line was crossed to the F1 generation, and the offspring was imaged (the transgenic Xenopus line is unpublished data from Schmucker’s lab).

## Additional files

Additional file 1. **Text S1.** Text file containing information about the background of the reconstruction, constraints for ALMOST, transparent objects, practical aspects, background of the imaging chamber and multicolor imaging, acquisition, reconstructing the 3D information, inversion, visualizing ALMOST datasets, and documentation. (PDF 617 kb)

Additional file 2: **Figure S1.** Simulation and illustration of imaging and reconstruction in OPT and ALMOST. (A) The processing in transmitted light OPT is depicted. An inverted (min and max values are swapped) Shepp-Logan Phantom, as used as standard in CT processing, is shown. Here, dark parts indicate absorbing parts in the sample. From this, transparent and absorbing sample projections are acquired, which form a sinogram. Using the standard software NRecon, the sinogram is inverted. Consequently, the sinogram can be reconstructed (calculation here done in MATLAB). For comparison, the inverted sample is shown. (B) Simulating ALMOST. We considered the outer and brightest ellipse in the Shepp-Logan phantom as the opaque surface of the sample, and thus, no information other than the first bright reflection is contributing to the image. The sample is illuminated with diffuse homogenous light against a white background. The surface will be visible. For an opaque sample only, the part facing the detector will be visible on the images. The second panel of B is illustrating this by showing only the upper half of the image. The third panel illustrates the parts contributing to the image when the sample is rotated by 60°. The camera is assumed to image from top. Projections from a series of images like the second and third panels create a sinogram from different angles of the sample. To mimic the processing of the use of the standard software NRecon, the sinogram is inverted (for simplicity only, max and min are swapped) and used for back projection with the same algorithm as above. This supports the idea that samples can be imaged and reconstructed with ALMOST akin to transmission images in the OPT. (C) Representation, depicting a multicolor object to demonstrate that the color appearance can be read out through individual color channels, where the differences in reflectivity will be imaged as intensity differences. (D) Schematic of the scenario if a semitransparent object is imaged. The gray ellipse surrounding the darker circle in the middle is considered semitransparent. In this case, theoretical information from the inside of the sample can be depicted. However, it is expected that due to multiple reflections at the interfaces, the intensity information will be difficult to interpret. It is of note that opaque parts within semitransparent samples will seem empty as no information from the inside is revealed like depicted in the schematic reconstruction in D. (TIF 8192 kb)

Additional file 3: **Figure S2.** Spectra of the used filters. An Amersham Bioscience (Amersham Pl Little Chalfont Buckinghamshire United Kingdom; now part of GE Healthcare (Chicago, Illinois, United States)) Ultrospec 2100 pro with Swift II software version 2.06 was used to acquire spectra of the three color filters used for three-color volume imaging. Spectra between 300 and 700 nm in 1 nm steps were acquired. Speed was 1800 nm/min; no reference was used. The transmission of the filters shows that, even with suboptimal filters, the color information can be retrieved. (PNG 235 kb)

Additional file 5: **Figure S3.** Automatic color balance. A) Photograph of a resistor. B) RGB reconstruction of the resistor using ALMOST, visualized in a projection. C) Same as in B with a surface rendering. Complementary colors are used for the visualization. The colors of the artificial surface rendering and light added to the rendered scene give a less vivid impression than the photograph or the projections but is a real 3D volumetric object with less transparency. D) Same as B and C but rendering performed with the open software Dristhi. Here, no inversion was performed after reconstruction with NRecon. Complementary colors were used. All channels were treated equally. The surface rendering in C and D differs due to the software used with colors being displayed slightly different. For the acquisition, an automatic white balance was performed using the Leica LAS software, which was driving the camera. Consequently, no individual adaptations for the different color channels have been performed. The overall contrast has been adjusted. This shows that an automated procedure can be used for the color balance in ALMOST. Scale bars = 500 μm. Imaging conditions are summarized in Additional file 20: Table S1. (PNG 1187 kb)

Additional file 6: **Figure S4.** Virtual sections through the seed cone sample (*Metasequoia glyptostroboides*) of Fig. 2J-O. A-C) transversal sections cutting perpendicular to the imaging axis. D-F) Frontal sections cutting at different places parallel to the focal plane. Scale bars = 500 μm. Imaging conditions are summarized in Additional file 20: Table S1. (PNG 2523 kb)

Additional file 8: **Figure S5.** Imaging glossy surfaces with ALMOST: the strongly reflective elytra of *Chrysolina americana* (Rosemary beetle) and a coin. A) Composite of three channels of raw images of the Rosemary beetle. B) Individual image from the ALMOST imaging (blue channel). C) Surface rendering of the beetle in 3D using three colors. D) The backside of a euroscent coin is shown. Scale bars = 2 mm. Imaging conditions are summarized in Additional file 20: Table S1. (PNG 814 kb)

Additional file 10: **Figure S6.** Performance of ALMOST and comparison with transmitted light OPT. We compared the imaging of a 400 copper mesh EM support grid in both modalities and reconstructed it. The two modalities pick up information through the holes of the mesh differently. A) Reconstructed transmitted light image of a 400 mesh TEM grid with 26 μm bars. B) Zoomed reconstructed transmitted light image. C) Zoomed reconstructed reflected light image. D) Overlay of B and C. To show the differences in the image formation between reflected and transmitted mode, we are showing a section through the grid for both modalities. E) Section through the reconstructed grid in transmitted light as indicated in A. Here, white indicates low transmission. F) Section through the reconstructed grid in reflected light. Here, white means high reflectivity. The image formation is cleaner for the transmitted light and some artifacts arise from specular reflection indicated by the thin diagonal dark lines. G) Reconstructed EM finder grid with letters in reflected light with 17 μm bars. Next, we imaged beads with an iron core of about 5 μm dispersed on a transparent coverslip. H) 5 μm Dyna beads, raw image acquired in the ALMOST device. I) Same Dyna beads as in H imaged with a Nikon C2 confocal microscope, × 20 objective with 0.75 NA. The ALMOST imaging can only detect the aggregates of the beads and is limited by the sampling of the camera (~ 4.2 μm per pixel in x,y and thus too coarse for picking up the small differences between the neighboring beads of 5 μm). J) *Drosophila* fly head. K) A virtual section is applied to J. L) Zoom of K as indicated by the rectangle in K. The characteristic curvature for the individual ommatidia of the compound eye becomes visible. M) Schematic of neighboring ommatidia. Drawing and size relations adapted from [58]. N) Intensity plot between two ommatidia as indicated by the red line in M in the region indicated in L. The line plot shows that the gap between the ommatidia can be visualized, and we, therefore, estimate that a difference of about 6–11 μm can be resolved. Scale bar in A, B, E, G, and K = 500 μm, in L = 100 μm, and in M = 10 μm. Imaging conditions are summarized in Additional file 20: Table S1. (PNG 4434 kb)

Additional file 11: **Figure S7.** Six channel spectral ALMOST imaging of a sea snail shell (*Pollia dorbignyi)*. A) Volume rendering of the mollusk shell side view using all six channels. B) Volume rendering of the shell bottom view; the shell is virtually cut open. C) Volume rendering of the shell, side view; the shell is virtually cut open. D) Six channel intensity distributions from the squared regions indicated by the arrows in A, B, and C. Differences in the spectral composition from the different regions can be revealed. The spectral specificity of the used filters is indicated by the colored bars; the line graph shows the spectral profile of the reflections from the different regions in the shell and the plasticine support. In the figures above, minor differences in the intensity between the different color channels were adapted manually. Here, for the six channels, the intensity of the background was kept constant to normalize for differences between channels. The intensity information from the different channels can be retrieved. The shell appears hollow as reflective light is imaged, which means that the light is blocked, thus not reaching the inner part, and no information is collected from the inside (see Additional file 2: Figure S1). Scale bar = 2 mm. Imaging conditions are summarized in Additional file 20: Table S1. (PNG 1352 kb)

Additional file 13: **Figure S8.** The 3D morphic potential of the *Drosophila* third instar larvae. A live larva was attached to an insect pin by adhesion. The larval body is in a contracted curled-up state when lifted from the ground. The larva was anesthetized with CO_2_ and kept in an atmosphere enriched for CO_2_ during imaging to prevent it from moving. Grayscale ALMOST is used to visualize the change in the outer shape of a larva. A) Difference between the same larva in a contracted (top) and a relaxed state (bottom). Exposure to 0.2 M NaN_3_ for 30 min induced the relaxed state. The arrows indicate the difference in length between the two states. Here, the induced relaxation shows that the larva is more stretched out (3705 μm) and longer than in the contracted and curled-up state (2648 μm). The difference in length between the contracted and the relaxed state corresponds to about 29% when measuring from rostral to caudal and about 13% when following the curvature of the contracted larva along the anterior-posterior axis (3035 μm vs. 3470 μm). B) Transversal cut through the larvae at the region indicated by the blue lines in A. The larva is oriented according to A, with the curled-up state on top. The black lines and arrows indicate the difference in the shape along the dextro-sinister (horizontal) axis of the larvae between the two states. The difference is 848 μm vs. 758 μm, corresponding to a difference of about 12%. C) Changes in the larva shape along the dorsoventral axis in the same region as indicated in A with the contracted state being left of the relaxed state. Interestingly, this difference is more pronounced than in the transversal axis (B). The difference between the two states along the dorsoventral axis is 932 μm vs. 758 μm, which amounts to a difference of 23%. Changes might be associated to specific pose. Scale bar = 500 μm. Imaging conditions are summarized in Additional file 20: Table S1. (PNG 605 kb)

Additional file 17: **Figure S9.** *Xenopus tropicalis* development. Graylevel ALMOST imaging is used for visualizing the surface of different developmental stages of *Xenopus tropicalis* embryos. 3D rendering of A) one-cell stage (stage 1), B) four-cell stage (stage 3), C) blastula stage (stage 7), D) large yolk plug stage (stage 11), E) neural plate stage (stage 14), F) mid neural fold stage (stage 15), G) an early tailbud stage (stage 25), and H) a tailbud stage (stage 28). Scale bars = 500 μm. Imaging conditions are summarized in Additional file 20: Table S1. (PNG 711 kb)

Additional file 19: **Figure S10.** A semitransparent technical object imaged by ALMOST. A) Raw Image of a LED. B) 3D projection using ALMOST revealing the outer shape. C) Cut view revealing parts from the inside of the LED. D-F) Zoomed images corresponding to the red rectangle indicated in A-C. B, C, E, and F are displayed using a color look-up table ranging from blue over yellow and white to orange. Scale bar = 500 μm. Imaging conditions are summarized in Additional file 20: Table S1. (PNG 4549 kb)

Additional file 20: **Table S1.** Table describing the imaging conditions per Figure. (PDF 354 kb)

Additional file 21: **Table S2.** Table containing details about the visualization. (PDF 343 kb)
