# Supplementary material for: A Label-free Multicolor Optical Surface Tomography (ALMOST) imaging method for nontransparent 3D samples
Source: BMC Biol. 2019 Jan 7;17:1. doi: 10.1186/s12915-018-0614-4 (PMC6323867; doi:10.1186/s12915-018-0614-4)
Supplement: Supplementary file 9 — Text S1. Text file containing information about the background of the reconstruction, constraints for ALMOST, transparent objects, practical aspects, background of the imaging chamber and multicolor imaging, acquisition, reconstructing the 3D information, inversion, visualizing ALMOST datasets, and documentation. (PDF 617 kb) [file 12915_2018_614_MOESM1_ESM.pdf]

Additional file 1: Supplementary text file

A Label-free Multicolor Optical Surface Tomography (ALMOST) imaging method for nontransparent 3D samples

Axelle Kerstens, Nikky Corthout, Benjamin Pavie, Zengjin Huang, Frank Vernaillen, Greetje Vande Velde, Sebastian Munck

1. Background of the reconstruction
2. Constraints for ALMOST
3. Semitransparent objects
4. Practical aspects
5. Background of the imaging chamber and multicolor imaging
6. Acquisition
7. Reconstructing the 3D information
8. Inversion
9. Visualizing ALMOST datasets
10. Documentation
11. References

1. Background of the reconstruction

We turned to existing tools to solve the problem of reconstructing 2D OPT reflected light (meaning ALMOST) images into a 3D surface representation of the sample [1], (Figure 1B). We theorized that this was possible given the fundamental similarities between our ALMOST approach and previously used transmitted light OPT, as well as CT. In all three cases, the device outputs a series of images collected at different angles. The mathematical foundation of the standard CT/transmitted light approaches are so-called line integrals, which represent the total attenuation of a straight ray traveling through the sample.

According to Beer's law, the total energy I_o emerging from the object along this ray is given by:

$$I_o = I_i e^{-\int \mu(x,y) ds} \quad (1)$$

with I_i being the total incident energy and $\mu(x,y)$ the local attenuation coefficient at (x,y) and the arc length s.

Beer's law is defined for homogenous distribution of an absorbing substance and for monochromatic light. It thus describes the transmission of the light through a homogenous sample with a material-specific attenuation coefficient. Similarly, the absorption of x-rays is related to the attenuating properties of the material a beam passes through. In reality, a sample can consist out of different materials having different attenuation properties. Therefore, in the context of CT imaging, typically $f(x,y)$ is used to describe a sample that attenuates x-rays passing through it with $\mu(x,y)$ being the attenuation of the material at position (x,y) within the sample. As such, the attenuation coefficient μ is responsible for the contrast of x-ray image dependent on the density of the materials in the sample that is imaged.

Thus considering the total attenuation along any line through the sample, the image formation process can be described by the Radon transform $P_\theta(r)$ of the object;

$$P_\theta(r) = \int_{-\infty}^{\infty} \int_{-\infty}^{\infty} f(x,y) \delta(x cos\theta + y sin\theta - r) dx dy \quad (2)$$

with δ being the Dirac delta function, r the perpendicular distance from the line to the origin, and θ the angle formed by the distance vector (Fig 1B) [2].

As the image formation is described by (1) and (2), consequently the inverse Radon transform needs to be performed to reconstruct the underlying 3D shape. Different practical solutions exist for image reconstruction. A back projection algorithm is typically used to reconstruct 3D objects. A simple discrete description of the filtered back projection algorithm for the inverse Radon transform of parallel projection data can be considered as;

$$f_{reconstructed}(x,y) = \frac{\pi}{N}\sum_{i=0}^{N-1} Q_{\theta_i}\,(xcos\theta_i + ysin\theta_i) \qquad (3)$$

with N being the number of projection angles and $Q_{\theta i}$ being the product of P_θ and the Radon projection kernel, filtered for data sparsity [1].

That means that in CT and OPT, using the filtered back projection algorithm, *f(x,y)* is estimated in 3D from the projections P_θ at the angles θ.

More sophisticated reconstruction algorithms exist, for example for low dosage imaging using dictionary learning [3], compressed sensing [4] or using the knowledge of the point spread function to improve the resolution of the reconstruction [5], but are not necessary for the considerations here, as the basic concepts still apply.

In ALMOST, different to transmitted light OPT, we are imaging reflected light. Imaging reflected light means that the reflectance (*R*) of the sample determines the image with *R* being typically approximated by:

$$R = \frac{I_{reflected}}{I_{incoming}} \qquad (4)$$

with $I_{reflected}$ being the intensity reflected for non-mirroring material with some roughness and $I_{incoming}$ being the intensity of the light source. Depending on the material, *R* typically changes with the wavelength of the light for colored samples, showing different degrees of reflectance for different wavelengths [7].

Different than in the case of absorption described above, the reflection needs to be considered directional depending on the illumination. If we consider Lambertian reflection, we can consider the reflection as the dot product of the surface's normal vector $\vec{N}$, and a normalized light-direction vector $\vec{L}$, pointing from the surface towards the light source. The amount of reflected light is then depending on the reflectivity R (in dependence of the wavelength, see (4)) of the surface and the intensity of the light hitting the surface:

$$I_{reflected} = \vec{L}\cdot\vec{N}RI_{incoming}, \qquad (5)$$

which is corresponding to Lambert's cosine law, saying that the radiant or luminous intensity emitted from a diffuse reflecting surface is direct proportional to the cosine of the angle between the direction of the incident light and the surface normal.

We further theorized that we could decode the $I_{reflected}$ into an image of the sample, displaying the shape and surface properties of the sample by the light detected. Using the 3D

capability of the OPT device together with homogenous illumination from the direction of the acquisition and acquiring multiple images of a sample as it rotates relative to the detector, we could then acquire a stack of images displaying a discrete 360° view of the sample akin to absorption OPT. Therefore we could formulate (2) for the reflection case imaged in an OPT correspondingly as:

$$P'_\theta(r) = \int_{-\infty}^{\infty}\int_{-\infty}^{\infty} I_{reflected}(x,y)\delta(x\cos\theta + y\sin\theta - r)dxdy \qquad (6)$$

where $I_{reflected}(x,y)$ is defined as in (5) for all points *(x,y)* on the surface of the sample facing the light source ($\vec{N}\cdot\vec{L} > 0$), and where it is 0 elsewhere. This resembles algorithms used in computer graphics, where it is called "back face culling" and is implemented using that very same dot product [8].

For rotationally symmetrical (i.e., cylindrical) objects the dot product $\vec{N}\cdot\vec{L}$ in (5) is constant and $\frac{I_{reflected}}{I_{incoming}}$ is proportional to R. Equation (6) is then independent of the sample surface geometry and the back-projection algorithm yields the true reflectivity R. However, for arbitrary sample shapes the local surface geometry influences the intensity of the reflected light. This leads to aberrant intensity values being recovered, marking a difference between the absorbed (transmitted OPT) and reflected light OPT (ALMOST). In practice, we found that surfaces of convex objects can be reconstructed well (e.g., Figure 2).

In any case given that the sample receives constant homogeneous indirect diffuse illumination at all imaged angles θ from the direction of the acquisition and by using a bright background, the intensity of the background -*i.e.,* where there is no sample- remains constant. In contrast, the variation of the brightness information in the foreground, where the sample is depicted, corresponds mainly to the specific reflective properties of the sample at each angle.

Therefore, given a bright background, we then thought that a sample with reflective properties creates an image that provides a comparable input for the filtered back projection algorithm as a transmission image, from an absorbing sample.
Given the similarity between (6) and (2), we considered that (3) can be used to approximate the 3D surface of a sample imaged with ALMOST.

It is of note that the filtered back projection algorithm weighs the reconstruction towards high frequencies to correct brightness issues due to the discrete nature of the input. For ALMOST, this also supports the reconstruction, where brightness differences across different sides of the sample can exist.

Overall, the major difference for the theoretical background between transmitted OPT/CT imaging and our ALMOST reflected light mode is that the underlying quantity that generates the signal is changed and that the reflectivity (*R*) (4) replaces absorption (1) (Figure 1C and 1D) for the generation of the individual projections. The back-projection algorithm can similarly back-calculate the true 3D shape of the imaged object from a series of 2D images collected at different angles as it does for other imaging modes (transmitted light or fluorescent light) even with a changed input using reflected light (as opposed to the transmitted light). In other words, the reconstruction algorithm (4) operates independently to whether light originates from the surface of the object or behind it.

2. Constraints for ALMOST

With ALMOST, we aim to depict 3D surfaces. That means we are looking at interactions of light with matter. Light hitting a surface can be reflected in a mirror type of fashion, meaning specularly reflected, or it is scattered, meaning that it is reflected with diffuse scattering. The light can also be absorbed. Real live samples can exhibit a combination of these phenomena. The color appearance of a sample is determined by the wavelengths of light predominantly scattered, with the rest of the light being absorbed or transmitted. If objects scatter all wavelengths with roughly equal strength, they appear white. If they absorb all wavelengths, they appear black.

Here we describe ALMOST as a method allowing to retrieve color information of the sample. However, it should be mentioned that here we only looked at the visible optical spectrum. Given the optical design, several optical properties cannot be examined with the current implementation. The setup is imaging light reflected from the sample, therefore ALMOST is not accessing transmittance. Also, no refractive index can be measured. In addition, no polarization or dispersion properties can be examined, including pleochroism. Regarding dichroism, the differential reflection of different wavelengths should be possible to be retrieved as color information, however, no polarization based dichroisms are currently measurable. The intensities resulting from color dichroism will be difficult to interpret. Samples with iridescence (pearlescence) can be problematic as due to this effect the intensity of the sample easily exceeds the background intensity under specific angles, leading to difficulties in reconstruction and visualization. As with every optical setup, diffraction will influence the images. Here, small surface structures can create artifacts. Examples can be surface structures that act as optical grids or patterns that create moiré effects.

In samples that are milky, the multitude of geometric scattering events inside them, akin to an emulsion, can lead to attenuation of the signal. The reduced back reflected light will then be imaged in our setup. For this kind of samples, differences between background and scattering

material can be difficult to detect under dim conditions, analogous to the macroscopic phenomenon called flat light, where visual indicators like the horizon disappear, for example when traversing ice fields under a grey sky. Other effects like Rayleigh and Mie scattering or plasmonics have not been considered for ALMOST.

As such, the sample needs to be static during a rotation. It is expected that the sample is not creating lensing effects, as lensing and dispersion will cause artifacts in the reconstruction. Birefringence in samples likely also disturbs reconstructions but has not been explored. Ongoing emissive processes, including photoluminescence, like chemiluminescence, bioluminescence, and fluorescence, but also incandescence can influence the result and the measured intensities.

3. Semitransparent objects

Even though principally some information of (semi-) transparent objects can be retrieved (see Additional file 19: Figure S10), due to several geometrical reflections the quantitative interpretation can be difficult. Also, inner structures can only be visualized if the overall image of the sample already reveals the inner structures. This means that smaller changes *insid*e a nontransparent hull will not be visualized, while for example the metal parts inside the transparent plastic hull of the LED can be depicted. It is of note that the visualization, in this case, is not trivial for projections and cut views likely show internal structures better (see also Additional file 19: Figure S10). In addition, for (semi-) transparent samples no phase information and no dispersive properties of the object like described above can be retrieved. Like with opaque samples, currently, no polarization information can be measured.

4. Practical aspects

Samples should not be mirror-like objects, are assumed to have some amount of reflecting properties, and exhibit diffuse reflection. The sample needs to fit the field of view. If it slightly exceeds in one direction partial reconstructions are possible, however, it is advisable that samples are not bigger than the field of view. The illumination is assumed to be homogenous and non-directionally.

In ALMOST we are aiming for imaging diffuse reflection. The reason is that diffuse reflecting objects appear as being luminous in the sense that under these conditions the light radiates from the sample in different directions. Like this we capture light coming from the sample, which we use akin

to light being emitted from a cleared fluorescent sample or that is shining through a transparent sample.

5. Background of the imaging chamber and multicolor imaging

To mimic the case of an absorbing sample, intensities are visualized relative to a background, which is typically brighter than the sample. As such, the filtered back projection algorithm can reconstruct information that is dimmer than the background. In order to display the object in a projection using the common tools, the background needs to be rendered see-through. When the background intensity value is rendered see-through, this means similar intensity values are also see-through. Therefore, we are imaging relative to a bright background with the sample appearing relatively dimmer than the background. The sensitivity is thus dependent on the detector and relative to the background. The detector and used filters will define wavelength information.

For color reconstruction using several wavelengths, the background is crucial as well, as it allows normalizing to this reference. The background reference can then be used for what is called color or white balance in photography and histology.

This adjustment is necessary to make the different colors displayed on a reconstructed sample appear with the same as on the original sample. Therefore, it is important that the background reference has a neutral color so that it appears neutral in the reconstruction.

We used white paper for color balance. It is of note that white paper can be fluorescent and if the illumination is containing UV light, the paper will be more blueish. Therefore, attention should be paid to this effect, as otherwise, this leads to a yellow tinted appearance after color balance operations. It is of note that in photography a gray card is used as a reference (like for example the Kodak R-27 Gray Cards).

Overall, using a homogenous neutral background is thus a constraint for adjusting the channels for multicolor ALMOST imaging.

6. Acquisition

After sample placement, images are acquired. In our case we used the BIOPTONICS software, however, also other solutions to create a stack of images at different angles can be used [9, 10].

7. Reconstructing the 3D information

Once a stack of images is created, for the consequent reconstruction, we used NRecon from SkyScan (now Bruker micro-CT), which is used for micro-CT reconstruction and can be downloaded for academic use. While Nrecon is kind of a standard, other solutions exist (Including MATLAB and ImageJ solutions). In our hands, NRecon can be tricky to accept tif-files from generic solutions, and potential alternative programs or conversions for writing tifs need to be used. For the reconstruction itself, care needs to be taken that the whole information is taken into account for the reconstruction and that the borders of the histogram are not trimmed (see 10. Documentation below).

8. Inversion

As NRecon is originally meant for reconstructing CT (and later for OPT) data, which are originating from x-ray imaging, where x-rays were traditionally used to blacken film. That means that the light parts were then used for displaying the absorbing parts, namely the bones. This type of inversion is kept for CT imaging (see the Shepp-Logan Phantom) and is used in NRecon. Practically, it means that the data is inverted during the reconstruction. NRecon applies an operation, where the intensity value is converted

value = ln(I0/I)),

with I being the intensity value of the projections images acquired in the CT/OPT/ALMOST device and I0 is the reference (air) intensity in the CT, the white background in the OPT and ALMOST. (See the NRecon software manual 2016 for more information.)

However, from a light imaging perspective, this inversion is counterintuitive, and for color display, it will return the wrong colors. Therefore, the inversion needs to be rectified after reconstruction for visualization. If the intensity values for the different wavelengths need to be interpreted and as the logarithm is a nonlinear function, the exact inverse of the operation of NRecon needs to be performed. For purely displaying the sample, the max and the min of intensity scale can be swapped. However, the sample may appear to have the equivalent of a different gamma applied in the visualization (see below: 9. visualizing ALMOST datasets, 10. Documentation and Additional file 21: Table S2). Practically the effect on the visualization might be not very prominent as reversing the NRecon operation visually is relatively untellable from the swopped min - max visualization we used after general contrast adjustment (Additional file 5: Figure S3, Additional file 21: Table S2).

Alternatively, the data can be reconstructed with other solution including MATLAB and ImageJ solution (RadonJ) where the inversion can be changed or avoided.

9. Visualizing ALMOST datasets

As we use NRecon for reconstruction, the volume is created slice by slice as a stack that shows the information along the symmetry axis, *i.e.,* as a y-axis stack respective to the XY images acquired per angle. Consequently, there are different ways to visualize the information. The y-axis stack can be resliced from the top, and single projection can be made showing the sample from a fixed angle. This can be done easily in ImageJ/Fiji. For a continuous visualization, implementing a process called raytracing, different commercial and free solutions exist for that purpose, including Imaris, Arivis and Drishti.

As ALMOST reveals no inside information from opaque samples, it means that there is no information available from the inside for the visualization and therefore the inside will be see-through in a projection. This counterintuitive feature leads to the effect that structures from the front and backside can be visible in a projection (see Additional file 4: Movie S1).

In addition to projections, the imaged object can also be visualized using surface/volumetric rendering. For volumetric/surface rendering the object is visualized as a solid object, for a given threshold. Artificial light can then be used to create shading on these surfaces.

As NRecon introduces nonlinearity in the reconstruction through the inversion it applies (see above) and a logarithm is applied to the data during the reconstruction, this is a non-linear transform. For a realistic display of the color distribution, this needs to be corrected. Without mathematically undoing the inversion of NRecon and using Arivis only, in our hands, the best representation of the original colors is given using an inverted cubic representation of the gray levels for the maximum projection.

The way the rendering software displays the colors and how they combine different color channels, depends on the software. In our case, for the volumetric rendering, we had to use the complementary colors (*i.e.* cyan, magenta and yellow instead of red, green and blue) for accurate color display as compared to projections, where the red green and blue color model can be used (Additional file 5: Figure S3).

For the actual display, if no normalization between the channels or color/white balance is applied, the channels can be set to display the white background as maximum in the visualization

software, which corresponds to the disclaimer on the images, that the channels were adjusted by hand.

In 10. Documentation, below, we describe the most important steps for reconstruction and visualization.

10. Documentation

The following guide instructs how to reconstruct and visualize the images, according to how we processed the images for this publication.

A. Reconstruction of the ALMOST images with NRecon

1. Start the NRecon software.
2. Choose the dataset you want to reconstruct (a window will open automatically).
3. Perform a preview in the Reconstruction wizard. This will allow you to change/fine-tune some of the default parameters for the reconstruction

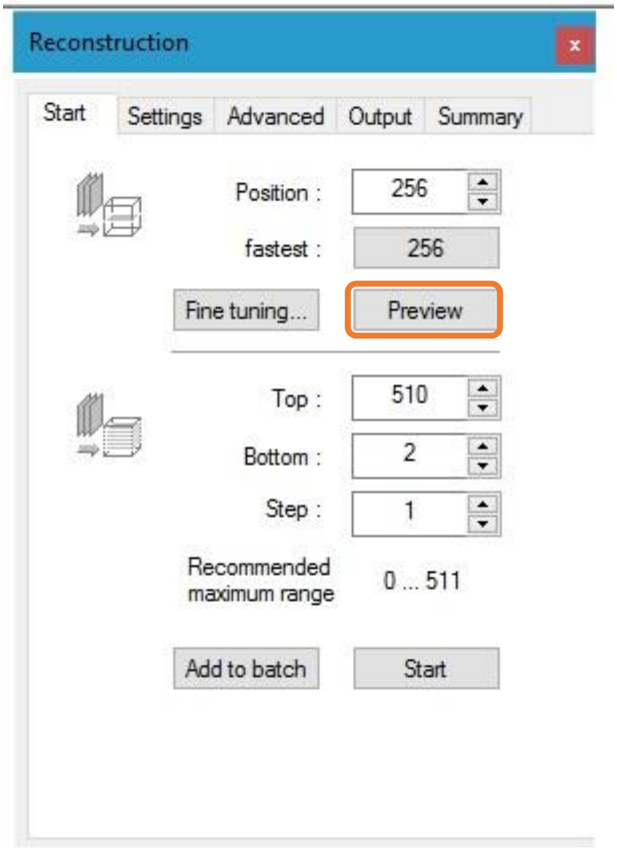

4. When clicking on ‘Fine-tuning’, a new window with the parameters for fine-tuning will appear, that allows to fine-tune acquisition parameters and correct for e.g., reconstruction artifacts in case they would appear. Press Start to run the fine-tuning with the number of trials you selected (5 by default).

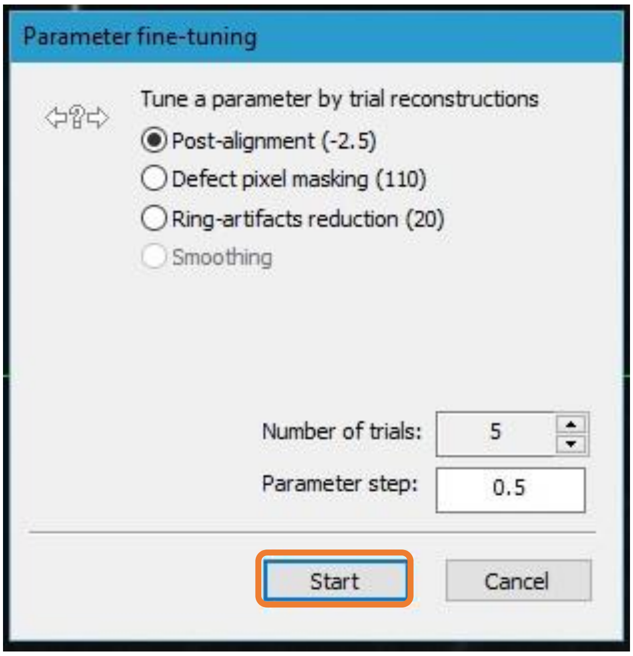

5. A message will appear, press OK.

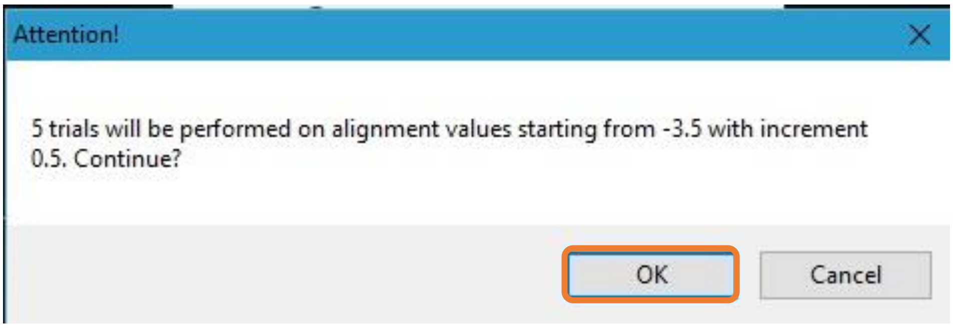

6. After preview or fine-tuning, the section Output will automatically be opened. Change the range of the histogram to ensure that it contains the peak and all the relevant data. Choose the optimal iteration from the fine-tuning by going through the trials with the arrows in the menu bar.
7. Deselect all the boxes for measurement scale, as they otherwise will appear in 3D reconstructions.
8. Choose the destination folder. (note: in the 'preferences' menu you can tick the option to automatically generate a destination folder for the reconstruction as sub-folder to the acquisition data)
9. Set the file format to TIF(16), keep the default parameters in the section Settings.
10. Go to the section Start.

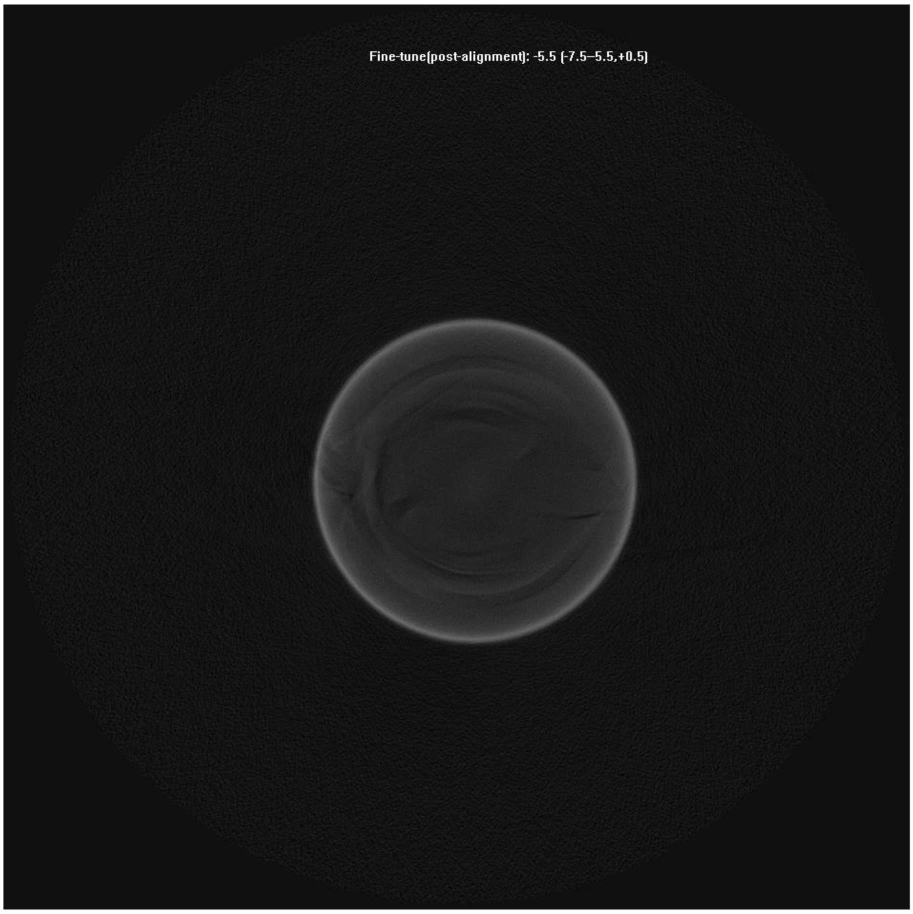

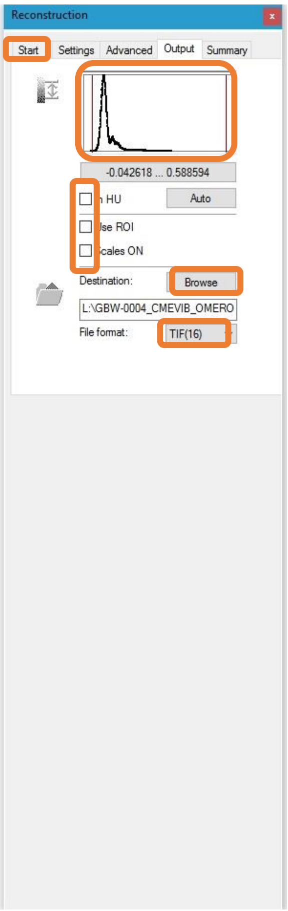

11. Press Start to initiate the reconstruction with all previously selected settings.

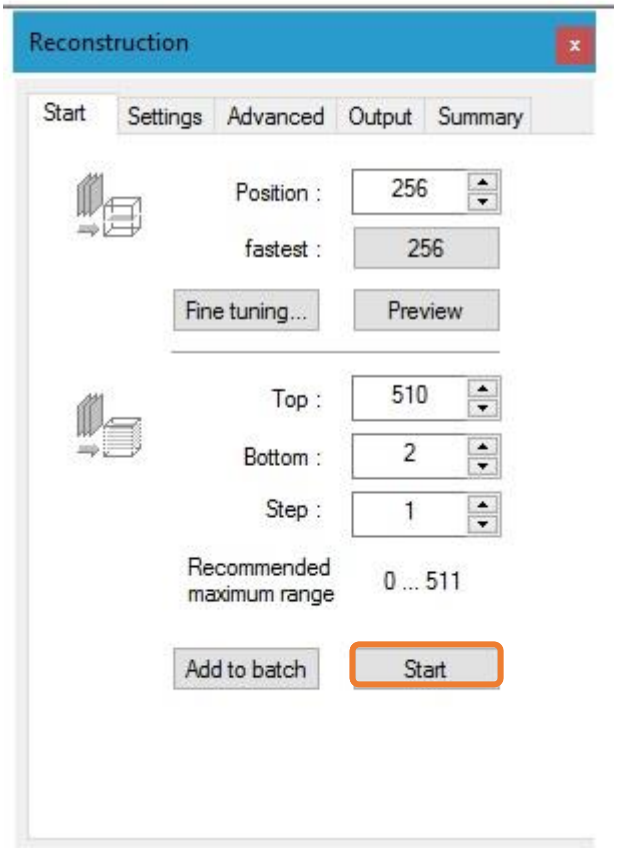

12. This can be repeated for the different channels (red, green, blue, fluorescent, …) or the different channels can be processed in batch.

B. 3D visualization in Arivis

1. Create image sequence stacks from the single tiff files via ImageJ.
2. Start the Arivis software and drag and drop the stacks in the GUI.
3. Choose Z-stacks as channels, select the folder where you want to save the .sis file and fill in the name of choice.

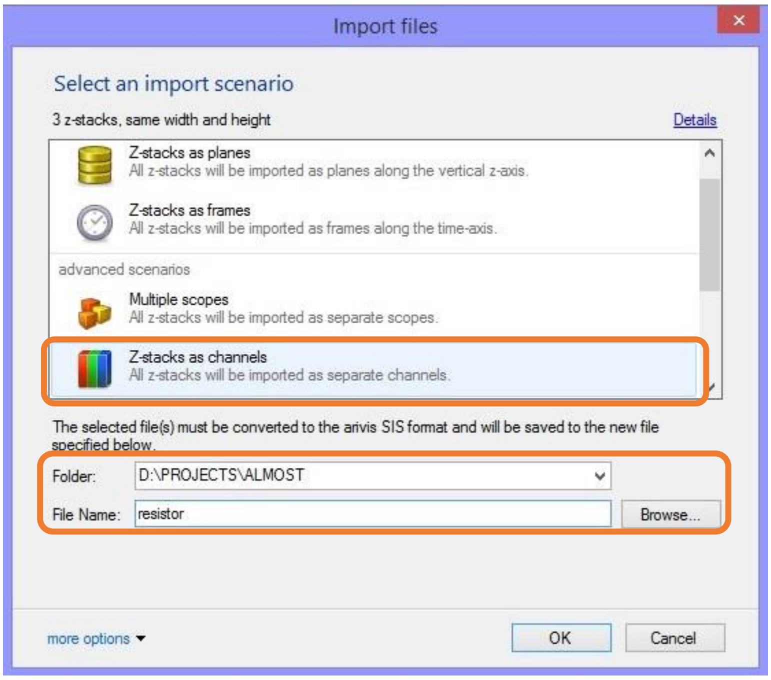

4. Choose the render mode bottom left of the screen. Maximum Intensity makes the objects appear to be transparent and mixes the channels (thus colors) 100%. In Volumetric you can choose the percentage of the channel mixing, the object is displayed as non-transparent.

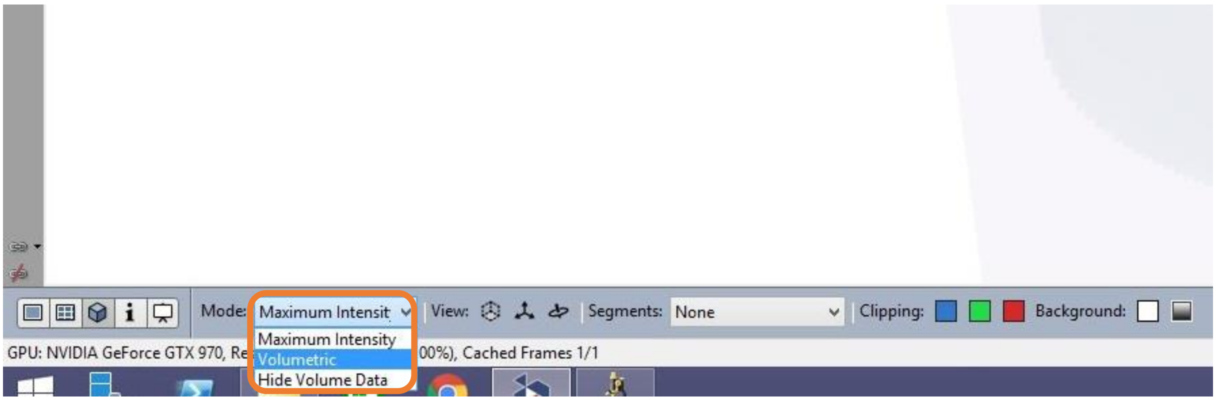

5.

5.1. Change the colors of the channels to the appropriate ones; you can do this by right-clicking the colored long vertical rectangles and selecting the color you want. In maximum projection mode, you should use red, green and blue. In volumetric mode you should display the red channel as cyan, the green channel as magenta and the blue channel as yellow.

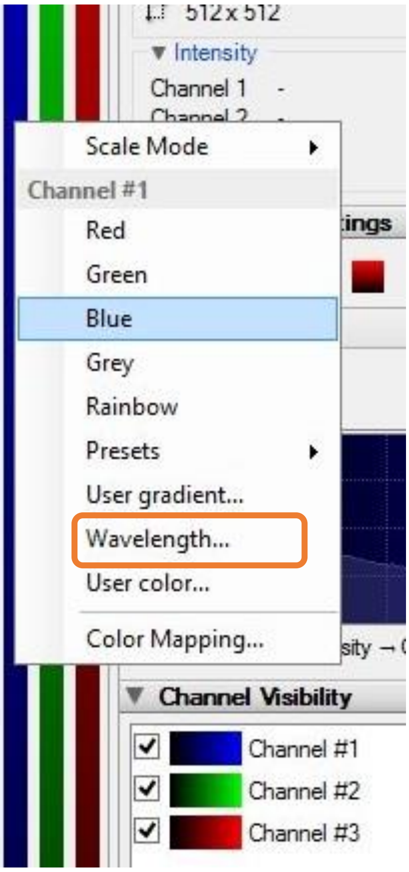

5.2. Instead of choosing a color, there is as well the option to choose a wavelength.

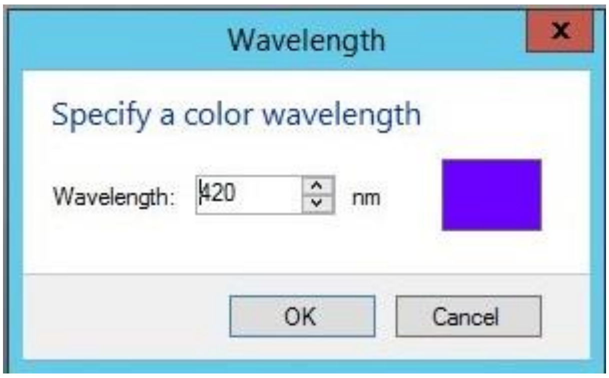

5.3. Apart from solid colors, it is also possible to display your data in gradients.

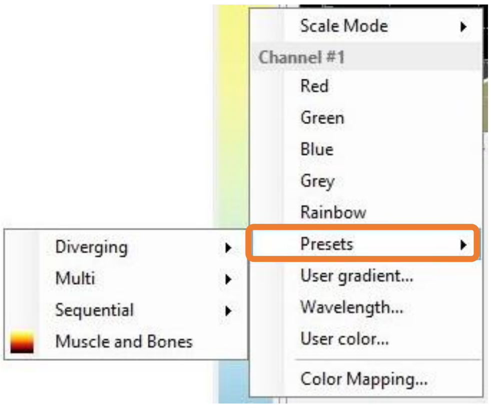

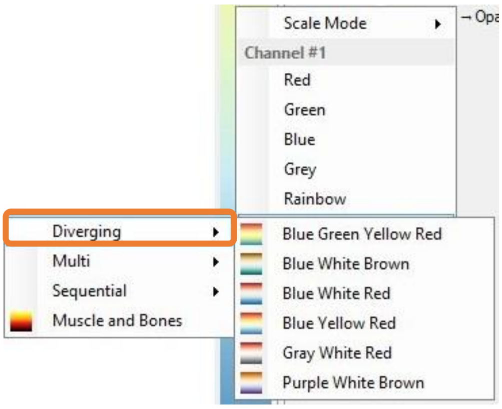

6. In 4D channel settings, you should select a preset in the intensity-opacity curve. In maximum intensity mode, the preset maximum intensity usually looks the best. By sliding the curve to the left or the right you can choose the intensity values you want to display. In volumetric mode the preset volumetric usually looks the best. To make sure that the colors mix (and not only cyan, magenta and yellow are visible) you should lower the opacity in every channel.

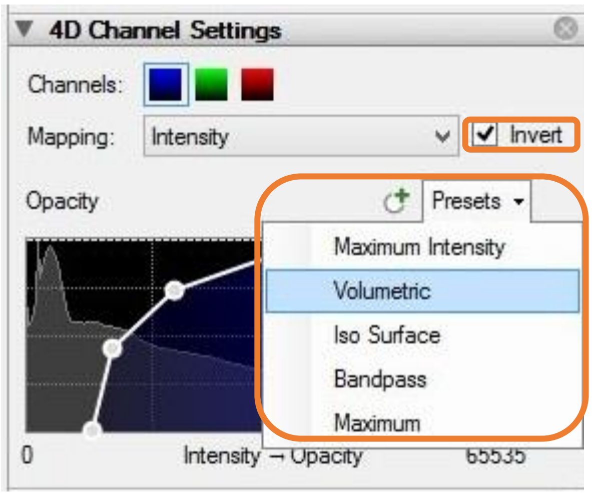

7.

7.1. Invert all channels that contain reflected surface information by ticking the box.

7.2. In case RGB colors are used, it is as well possible to invert the channels via the color curves. Use negative linear or mirrored cubic.

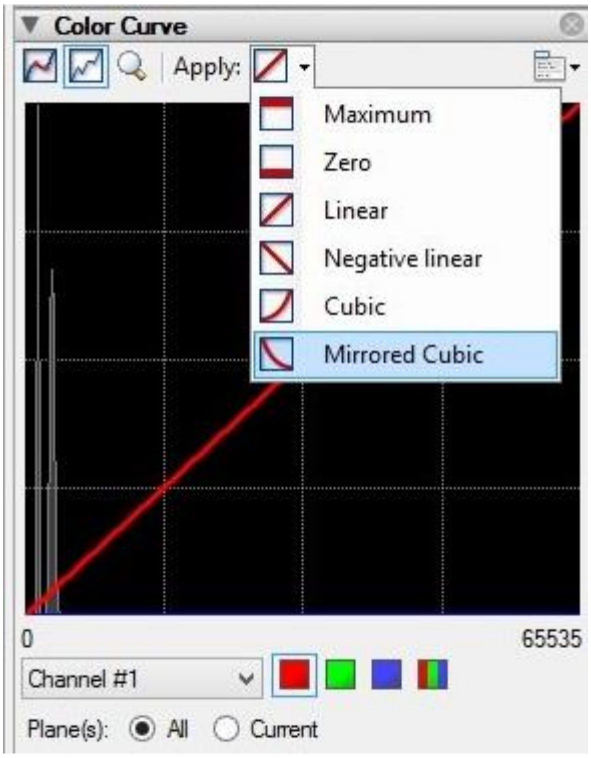

8. Adjust the overall view with the parameters defined in color sliders and 4D lighting and projection.

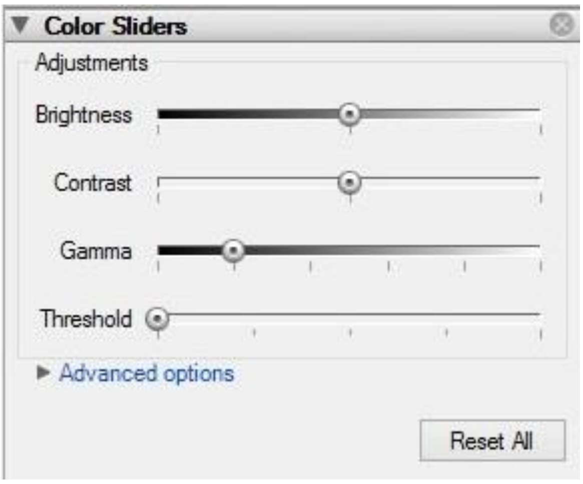

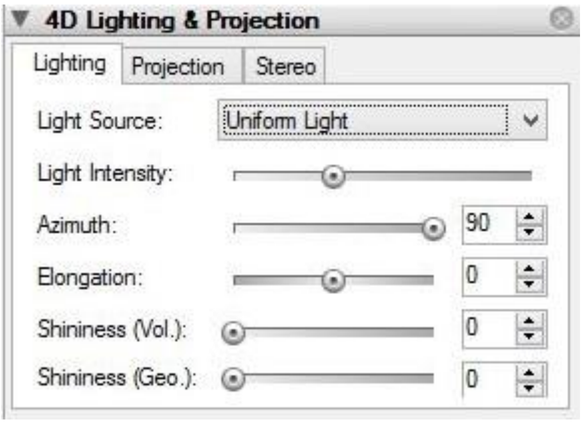

11. References

1. Kak AC, Slaney M: **Principles of computerized tomographic imaging**. Philadelphia: Society for Industrial and Applied Mathematics; 2001.
2. Semmlow JL, Griffel B: **Biosignal and medical image processing**, Third edition. edn. Boca Raton: CRC Press, Taylor & Francis Group, CRC Press is an imprint of the Taylor & Francis Group, an Informa business; 2014.
3. Zhang C, Zhang T, Li M, Peng C, Liu Z, Zheng J: **Low-dose CT reconstruction via L1 dictionary learning regularization using iteratively reweighted least-squares**. *Biomed Eng Online* 2016, **15**(1):66.
4. Correia T, Lockwood N, Kumar S, Yin J, Ramel MC, Andrews N, Katan M, Bugeon L, Dallman MJ, McGinty J, Frankel P, French PM, Arridge S: **Accelerated Optical Projection Tomography Applied to In Vivo Imaging of Zebrafish**. *PloS one* 2015, **10**(8):e0136213.
5. Trull AK, van der Horst J, Palenstijn WJ, van Vliet LJ, van Leeuwen T, Kalkman J: **Point spread function based image reconstruction in optical projection tomography**. *Physics in medicine and biology* 2017, **62**(19):7784-7797.
6. Pan XC, Sidky EY, Vannier M: **Why do commercial CT scanners still employ traditional, filtered back-projection for image reconstruction?** *Inverse Probl* 2009, **25**(12).
7. Hecht E: **Optics**, 4th edn. Reading, Mass.: Addison-Wesley; 2002.
8. Hughes JF: **Computer graphics : principles and practice**, Third edition. edn. Upper Saddle River, New Jersey: Addison-Wesley; 2014.
9. Gualda EJ, Vale T, Almada P, Feijo JA, Martins GG, Moreno N: **OpenSpinMicroscopy: an open-source integrated microscopy platform**. *Nature methods* 2013, **10**(7):599-600.
10. Watson T, Andrews N, Davis S, Bugeon L, Dallman MD, McGinty J: **OPTiM: Optical projection tomography integrated microscope using open-source hardware and software**. *PloS one* 2017, **12**(7):e0180309.
