# Supplementary figures and images for: A Label-free Multicolor Optical Surface Tomography (ALMOST) imaging method for nontransparent 3D samples
Source: BMC Biol. 2019 Jan 7;17:1. doi: 10.1186/s12915-018-0614-4 (PMC6323867; doi:10.1186/s12915-018-0614-4)

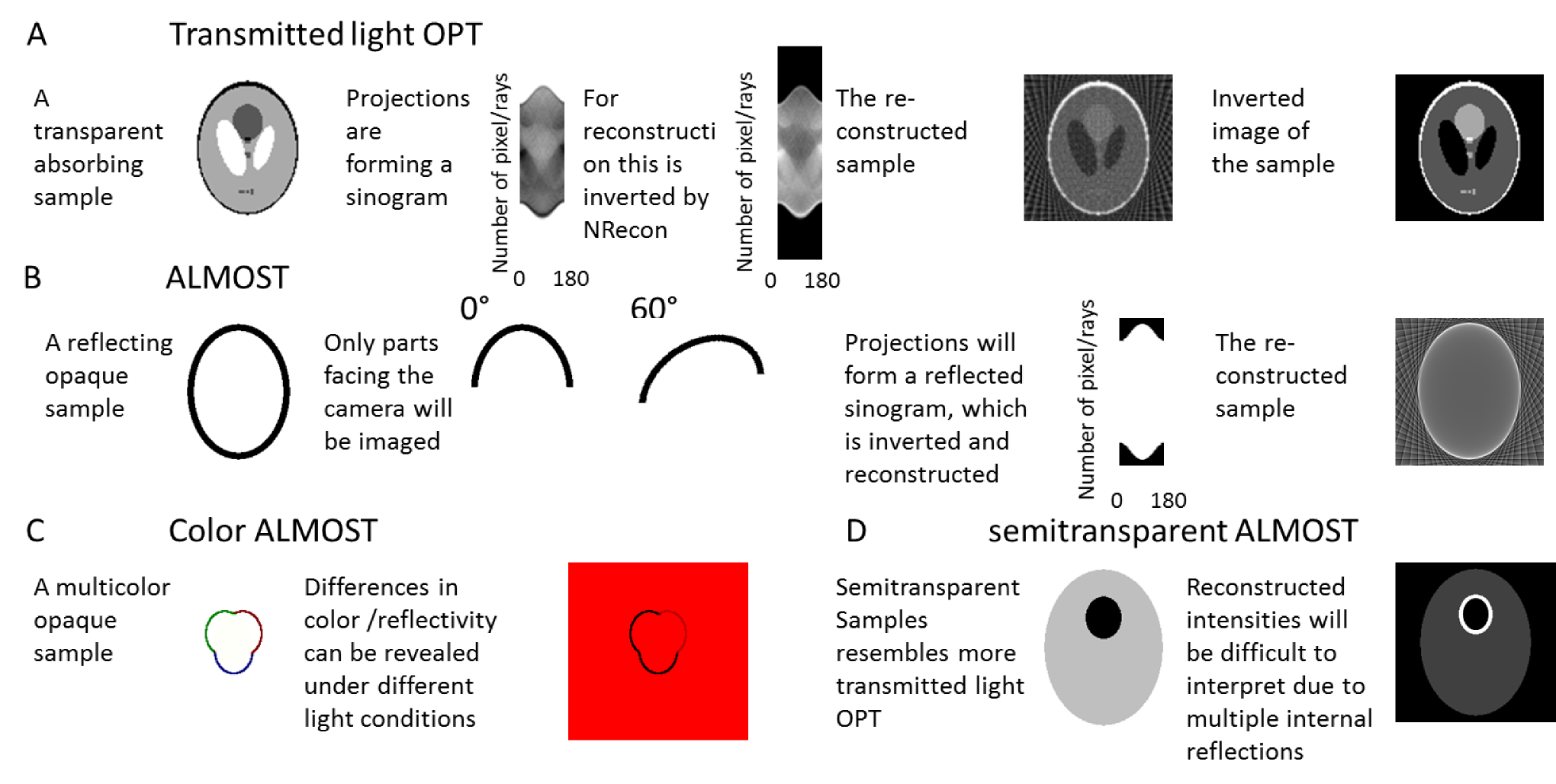

Supplement: Supplementary file 10 — Figure S1. Simulation and illustration of imaging and reconstruction in OPT and ALMOST. (A) The processing in transmitted light OPT is depicted. An inverted (min and max values are swapped) Shepp-Logan Phantom, as used as standard in CT processing, is shown. Here, dark parts indicate absorbing parts in the sample. From this, transparent and absorbing sample projections are acquired, which form a sinogram. Using the standard software NRecon, the sinogram is inverted. Consequently, the sinogram can be reconstructed (calculation here done in MATLAB). For comparison, the inverted sample is shown. (B) Simulating ALMOST. We considered the outer and brightest ellipse in the Shepp-Logan phantom as the opaque surface of the sample, and thus, no information other than the first bright reflection is contributing to the image. The sample is illuminated with diffuse homogenous light against a white background. The surface will be visible. For an opaque sample only, the part facing the detector will be visible on the images. The second panel of B is illustrating this by showing only the upper half of the image. The third panel illustrates the parts contributing to the image when the sample is rotated by 60°. The camera is assumed to image from top. Projections from a series of images like the second and third panels create a sinogram from different angles of the sample. To mimic the processing of the use of the standard software NRecon, the sinogram is inverted (for simplicity only, max and min are swapped) and used for back projection with the same algorithm as above. This supports the idea that samples can be imaged and reconstructed with ALMOST akin to transmission images in the OPT. (C) Representation, depicting a multicolor object to demonstrate that the color appearance can be read out through individual color channels, where the differences in reflectivity will be imaged as intensity differences. (D) Schematic of the scenario if a semitransparent object is imaged. The gray [file 12915_2018_614_MOESM2_ESM.tif]

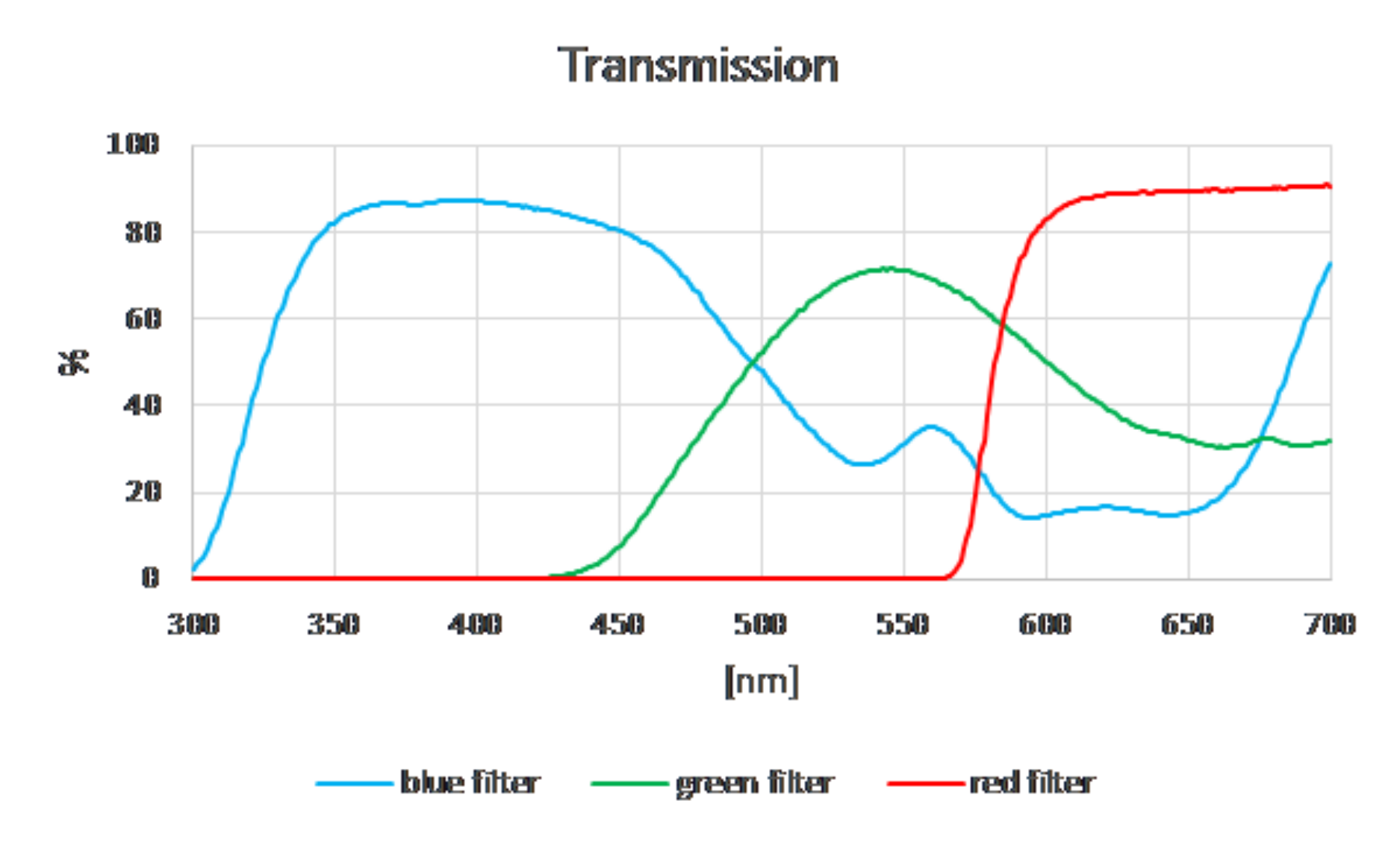

Supplement: Supplementary file 11 — Figure S2. Spectra of the used filters. An Amersham Bioscience (Amersham Pl Little Chalfont Buckinghamshire United Kingdom; now part of GE Healthcare (Chicago, Illinois, United States)) Ultrospec 2100 pro with Swift II software version 2.06 was used to acquire spectra of the three color filters used for three-color volume imaging. Spectra between 300 and 700 nm in 1 nm steps were acquired. Speed was 1800 nm/min; no reference was used. The transmission of the filters shows that, even with suboptimal filters, the color information can be retrieved. (PNG 235 kb) [file 12915_2018_614_MOESM3_ESM.png]

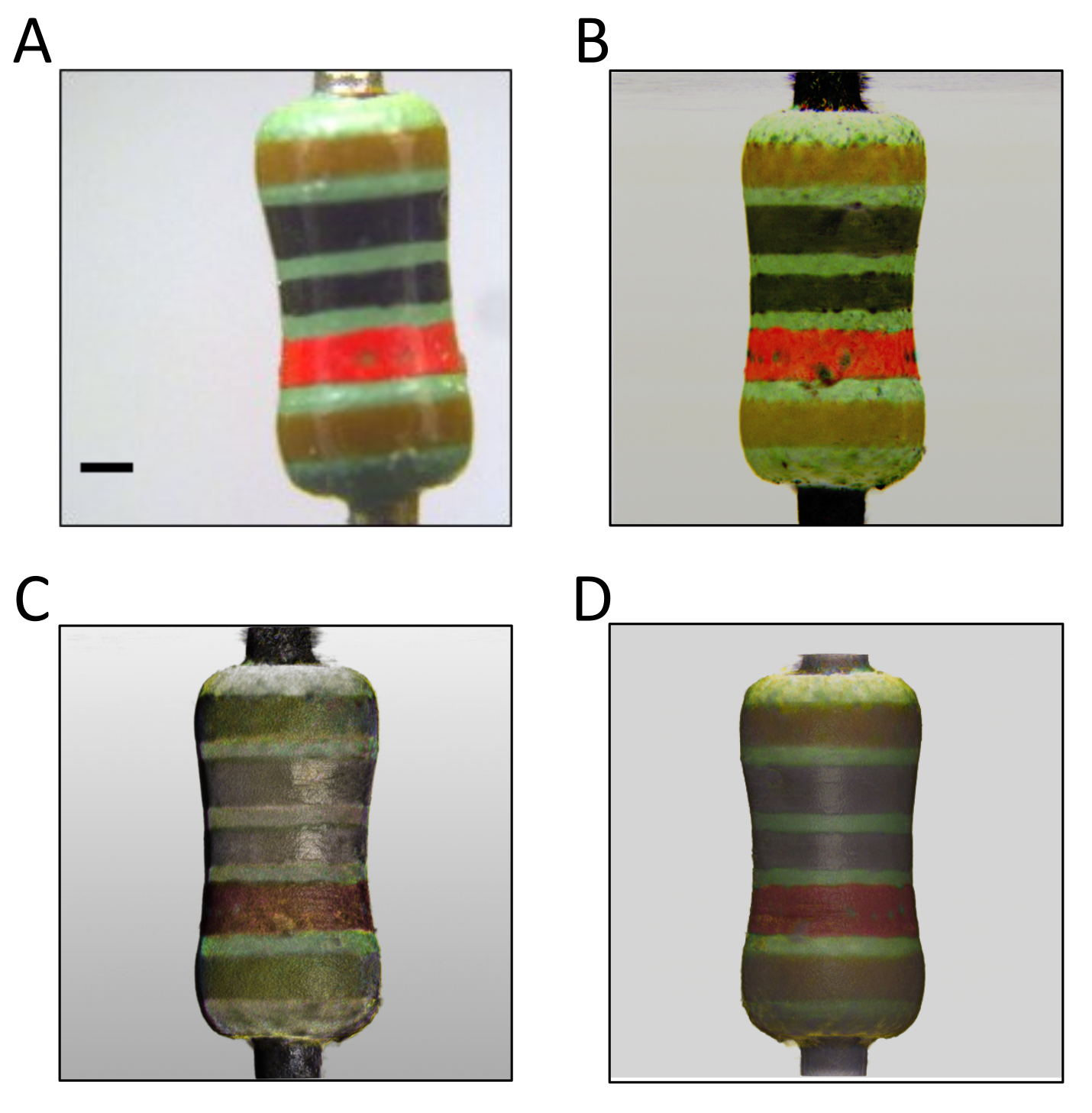

Supplement: Supplementary file 12 — Figure S3. Automatic color balance. A) Photograph of a resistor. B) RGB reconstruction of the resistor using ALMOST, visualized in a projection. C) Same as in B with a surface rendering. Complementary colors are used for the visualization. The colors of the artificial surface rendering and light added to the rendered scene give a less vivid impression than the photograph or the projections but is a real 3D volumetric object with less transparency. D) Same as B and C but rendering performed with the open software Dristhi. Here, no inversion was performed after reconstruction with NRecon. Complementary colors were used. All channels were treated equally. The surface rendering in C and D differs due to the software used with colors being displayed slightly different. For the acquisition, an automatic white balance was performed using the Leica LAS software, which was driving the camera. Consequently, no individual adaptations for the different color channels have been performed. The overall contrast has been adjusted. This shows that an automated procedure can be used for the color balance in ALMOST. Scale bars = 500 μm. Imaging conditions are summarized in Additional file 20: Table S1. (PNG 1187 kb) [file 12915_2018_614_MOESM5_ESM.png]

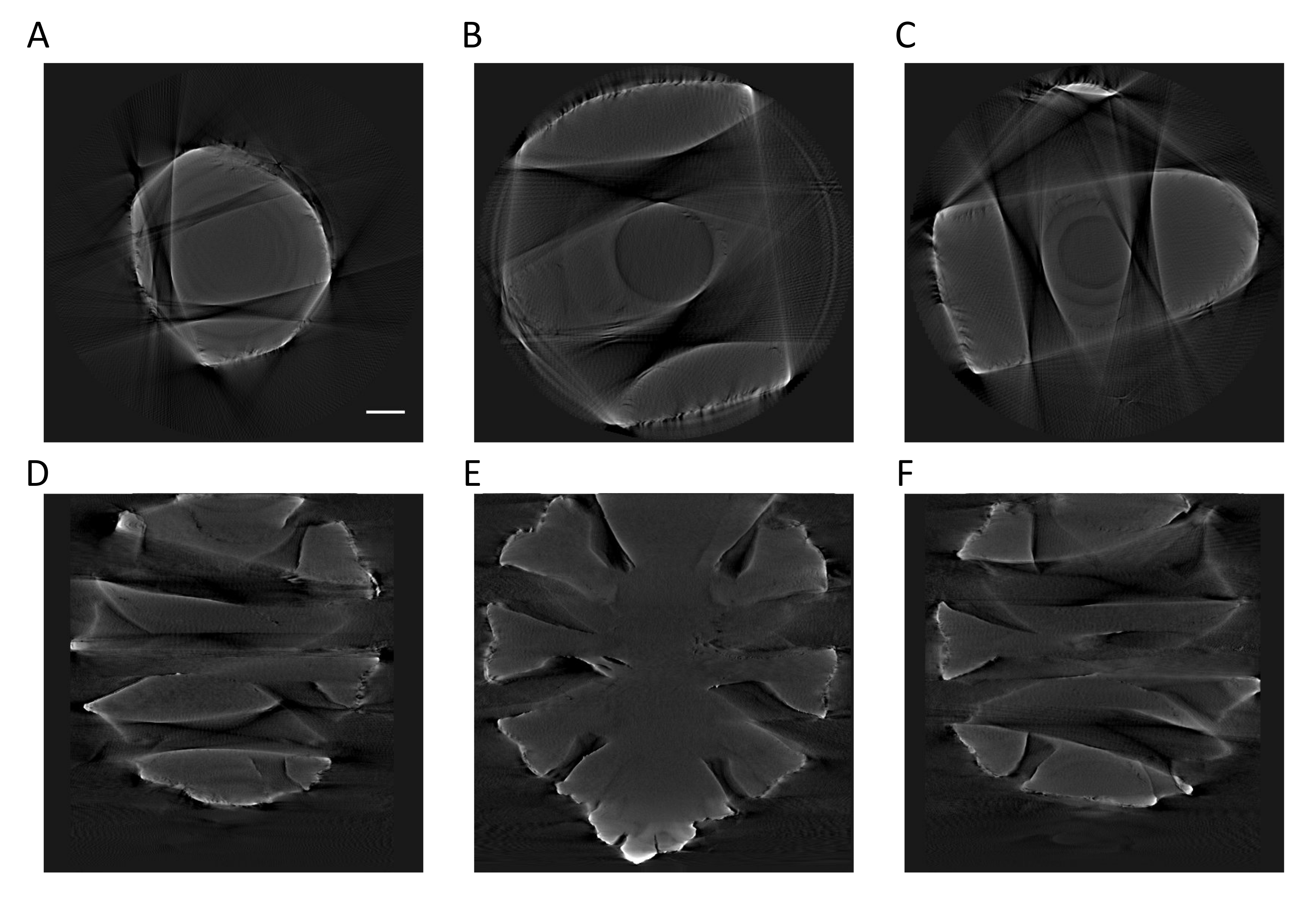

Supplement: Supplementary file 13 — Figure S4. Virtual sections through the seed cone sample (Metasequoia glyptostroboides) of Fig. 2J-O. A-C) transversal sections cutting perpendicular to the imaging axis. D-F) Frontal sections cutting at different places parallel to the focal plane. Scale bars = 500 μm. Imaging conditions are summarized in Additional file 20: Table S1. (PNG 2523 kb) [file 12915_2018_614_MOESM6_ESM.png]

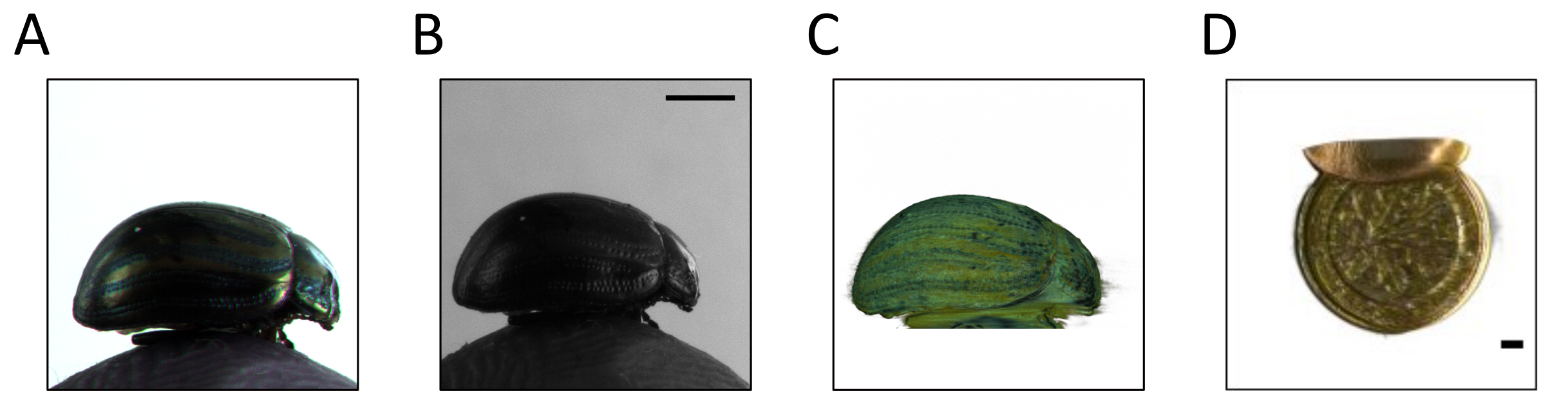

Supplement: Supplementary file 14 — Figure S5. Imaging glossy surfaces with ALMOST: the strongly reflective elytra of Chrysolina americana (Rosemary beetle) and a coin. A) Composite of three channels of raw images of the Rosemary beetle. B) Individual image from the ALMOST imaging (blue channel). C) Surface rendering of the beetle in 3D using three colors. D) The backside of a euroscent coin is shown. Scale bars = 2 mm. Imaging conditions are summarized in Additional file 20: Table S1. (PNG 814 kb) [file 12915_2018_614_MOESM8_ESM.png]

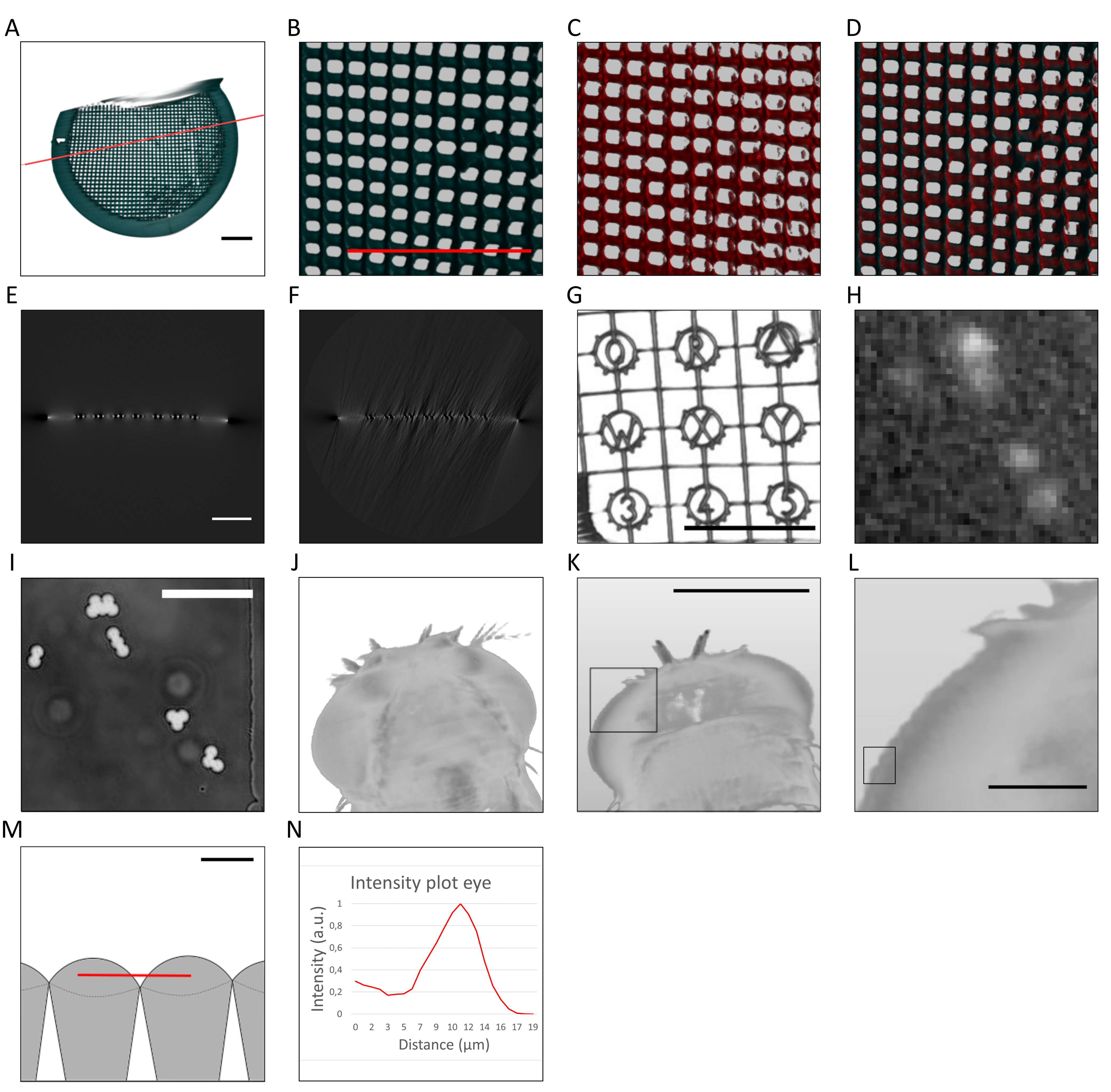

Supplement: Supplementary file 15 — Figure S6. Performance of ALMOST and comparison with transmitted light OPT. We compared the imaging of a 400 copper mesh EM support grid in both modalities and reconstructed it. The two modalities pick up information through the holes of the mesh differently. A) Reconstructed transmitted light image of a 400 mesh TEM grid with 26 μm bars. B) Zoomed reconstructed transmitted light image. C) Zoomed reconstructed reflected light image. D) Overlay of B and C. To show the differences in the image formation between reflected and transmitted mode, we are showing a section through the grid for both modalities. E) Section through the reconstructed grid in transmitted light as indicated in A. Here, white indicates low transmission. F) Section through the reconstructed grid in reflected light. Here, white means high reflectivity. The image formation is cleaner for the transmitted light and some artifacts arise from specular reflection indicated by the thin diagonal dark lines. G) Reconstructed EM finder grid with letters in reflected light with 17 μm bars. Next, we imaged beads with an iron core of about 5 μm dispersed on a transparent coverslip. H) 5 μm Dyna beads, raw image acquired in the ALMOST device. I) Same Dyna beads as in H imaged with a Nikon C2 confocal microscope, × 20 objective with 0.75 NA. The ALMOST imaging can only detect the aggregates of the beads and is limited by the sampling of the camera (~ 4.2 μm per pixel in x,y and thus too coarse for picking up the small differences between the neighboring beads of 5 μm). J) Drosophila fly head. K) A virtual section is applied to J. L) Zoom of K as indicated by the rectangle in K. The characteristic curvature for the individual ommatidia of the compound eye becomes visible. M) Schematic of neighboring ommatidia. Drawing and size relations adapted from [58]. N) Intensity plot between two ommatidia as indicated by the red line in M in the region indicated in L. The line plot shows that the gap between the ommatidia can [file 12915_2018_614_MOESM10_ESM.png]

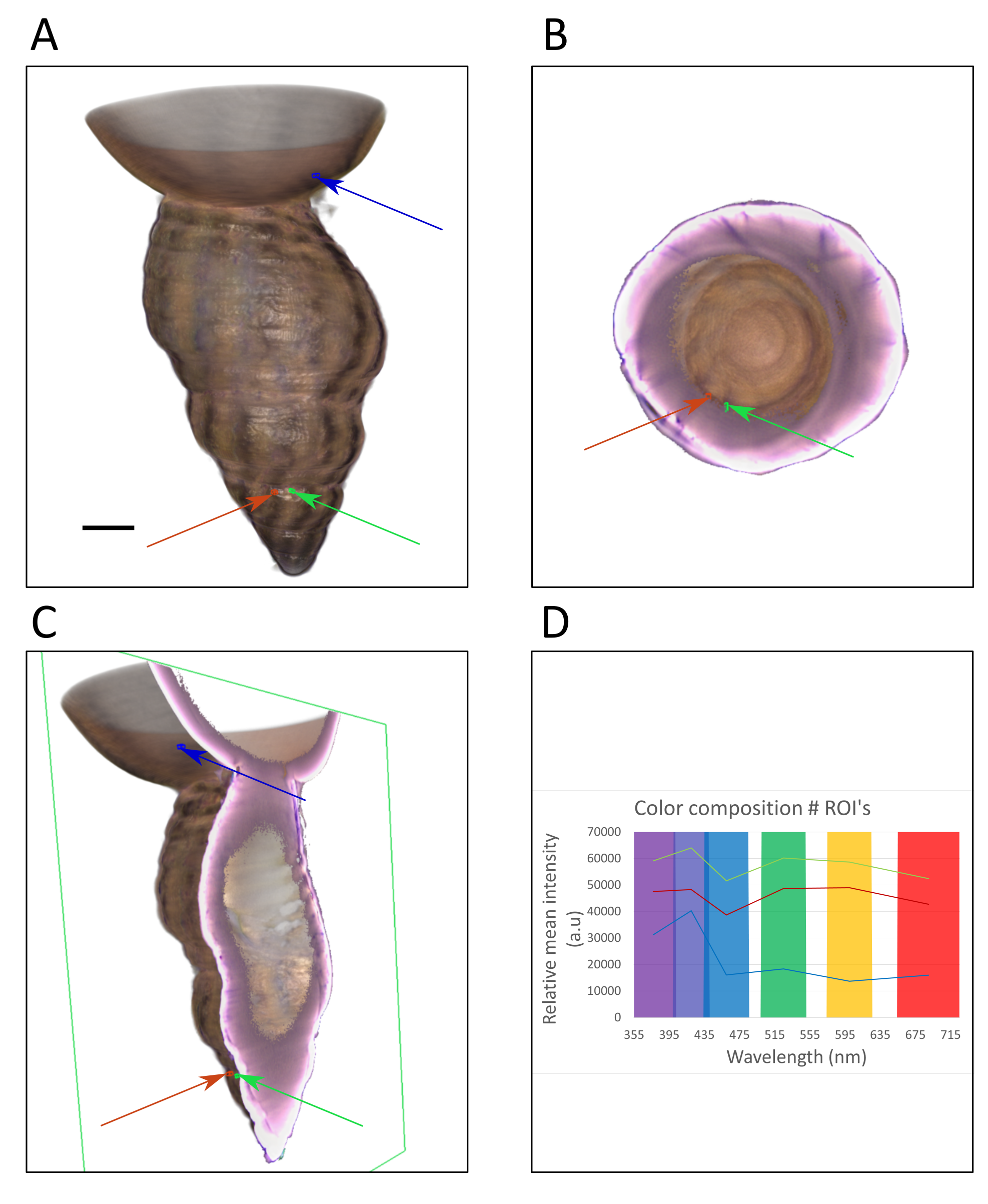

Supplement: Supplementary file 16 — Figure S7. Six channel spectral ALMOST imaging of a sea snail shell (Pollia dorbignyi). A) Volume rendering of the mollusk shell side view using all six channels. B) Volume rendering of the shell bottom view; the shell is virtually cut open. C) Volume rendering of the shell, side view; the shell is virtually cut open. D) Six channel intensity distributions from the squared regions indicated by the arrows in A, B, and C. Differences in the spectral composition from the different regions can be revealed. The spectral specificity of the used filters is indicated by the colored bars; the line graph shows the spectral profile of the reflections from the different regions in the shell and the plasticine support. In the figures above, minor differences in the intensity between the different color channels were adapted manually. Here, for the six channels, the intensity of the background was kept constant to normalize for differences between channels. The intensity information from the different channels can be retrieved. The shell appears hollow as reflective light is imaged, which means that the light is blocked, thus not reaching the inner part, and no information is collected from the inside (see Additional file 2: Figure S1). Scale bar = 2 mm. Imaging conditions are summarized in Additional file 20: Table S1. (PNG 1352 kb) [file 12915_2018_614_MOESM11_ESM.png]

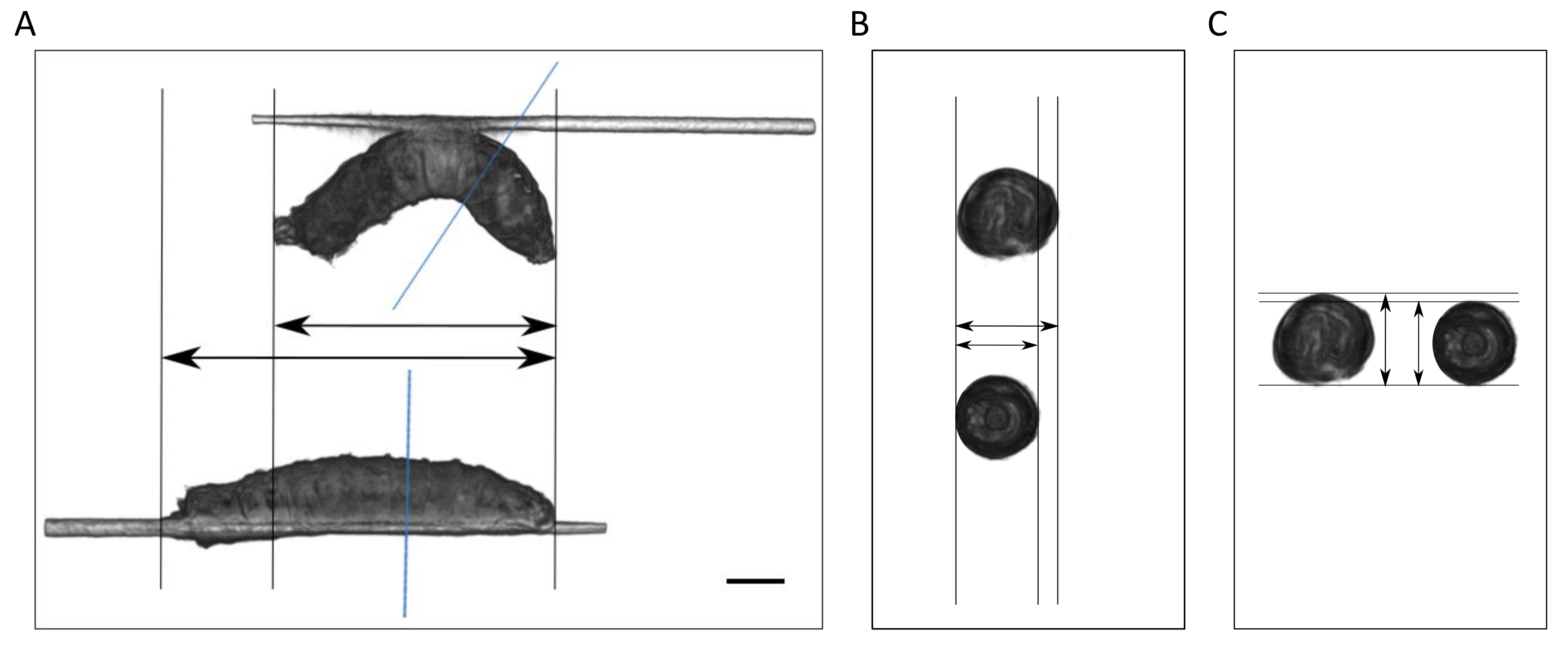

Supplement: Supplementary file 17 — Figure S8. The 3D morphic potential of the Drosophila third instar larvae. A live larva was attached to an insect pin by adhesion. The larval body is in a contracted curled-up state when lifted from the ground. The larva was anesthetized with CO2 and kept in an atmosphere enriched for CO2 during imaging to prevent it from moving. Grayscale ALMOST is used to visualize the change in the outer shape of a larva. A) Difference between the same larva in a contracted (top) and a relaxed state (bottom). Exposure to 0.2 M NaN3 for 30 min induced the relaxed state. The arrows indicate the difference in length between the two states. Here, the induced relaxation shows that the larva is more stretched out (3705 μm) and longer than in the contracted and curled-up state (2648 μm). The difference in length between the contracted and the relaxed state corresponds to about 29% when measuring from rostral to caudal and about 13% when following the curvature of the contracted larva along the anterior-posterior axis (3035 μm vs. 3470 μm). B) Transversal cut through the larvae at the region indicated by the blue lines in A. The larva is oriented according to A, with the curled-up state on top. The black lines and arrows indicate the difference in the shape along the dextro-sinister (horizontal) axis of the larvae between the two states. The difference is 848 μm vs. 758 μm, corresponding to a difference of about 12%. C) Changes in the larva shape along the dorsoventral axis in the same region as indicated in A with the contracted state being left of the relaxed state. Interestingly, this difference is more pronounced than in the transversal axis (B). The difference between the two states along the dorsoventral axis is 932 μm vs. 758 μm, which amounts to a difference of 23%. Changes might be associated to specific pose. Scale bar = 500 μm. Imaging conditions are summarized in Additional file 20: Table S1. (PNG 605 kb) [file 12915_2018_614_MOESM13_ESM.png]

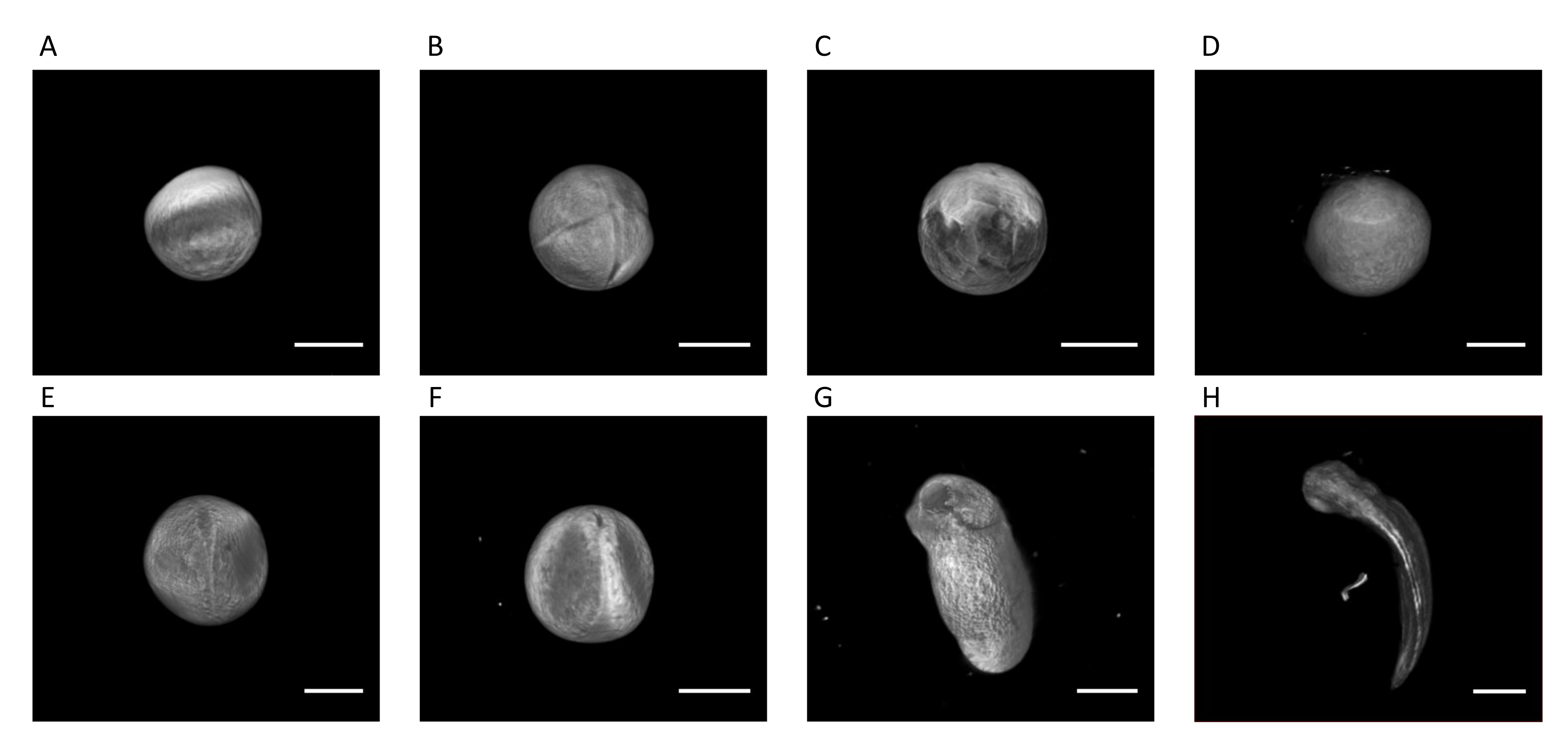

Supplement: Supplementary file 18 — Figure S9. Xenopus tropicalis development. Graylevel ALMOST imaging is used for visualizing the surface of different developmental stages of Xenopus tropicalis embryos. 3D rendering of A) one-cell stage (stage 1), B) four-cell stage (stage 3), C) blastula stage (stage 7), D) large yolk plug stage (stage 11), E) neural plate stage (stage 14), F) mid neural fold stage (stage 15), G) an early tailbud stage (stage 25), and H) a tailbud stage (stage 28). Scale bars = 500 μm. Imaging conditions are summarized in Additional file 20: Table S1. (PNG 711 kb) [file 12915_2018_614_MOESM17_ESM.png]

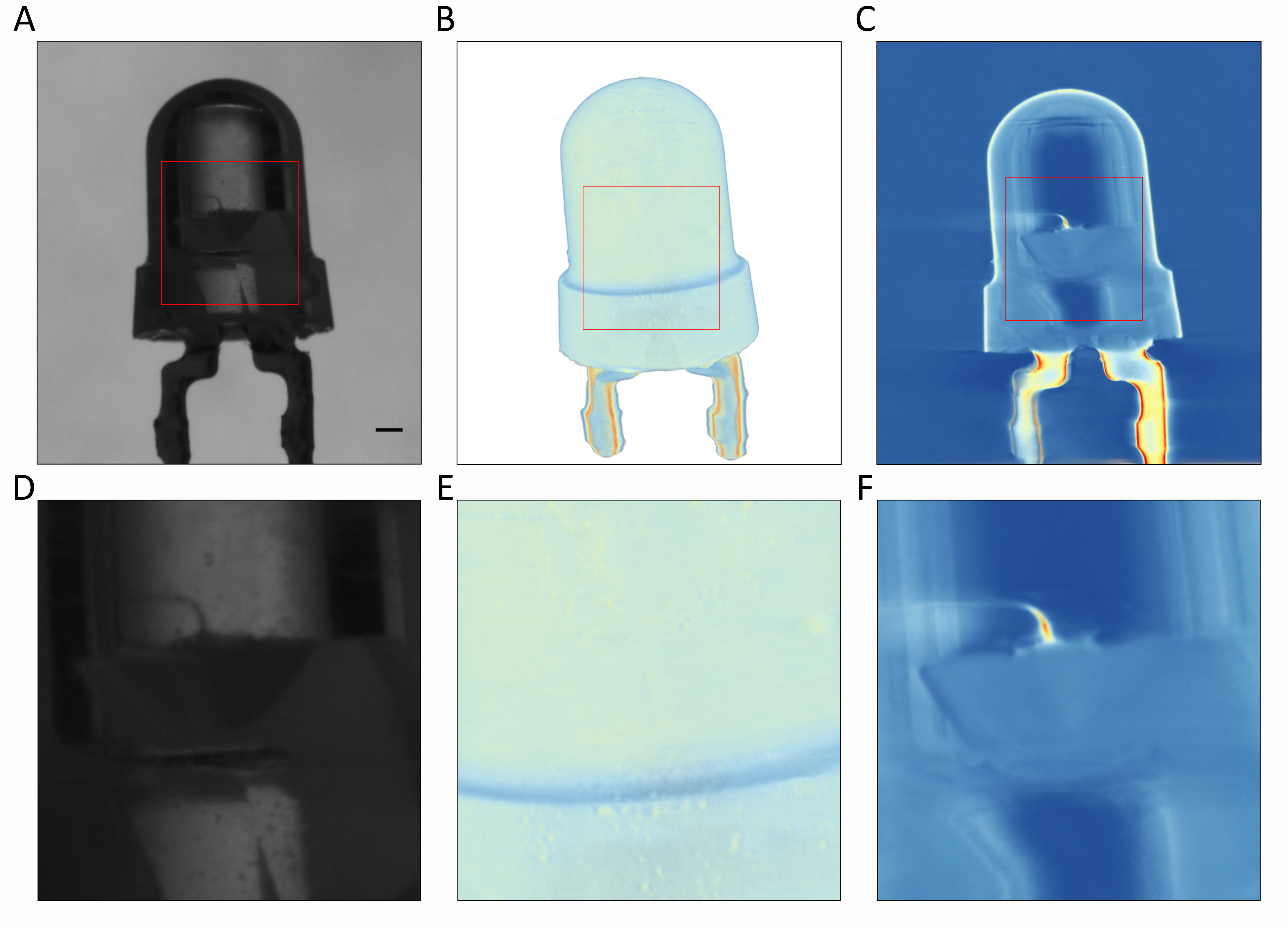

Supplement: Supplementary file 19 — Figure S10. A semitransparent technical object imaged by ALMOST. A) Raw Image of a LED. B) 3D projection using ALMOST revealing the outer shape. C) Cut view revealing parts from the inside of the LED. D-F) Zoomed images corresponding to the red rectangle indicated in A-C. B, C, E, and F are displayed using a color look-up table ranging from blue over yellow and white to orange. Scale bar = 500 μm. Imaging conditions are summarized in Additional file 20: Table S1. (PNG 4549 kb) [file 12915_2018_614_MOESM19_ESM.png]
