# Supplementary material for: A Label-free Multicolor Optical Surface Tomography (ALMOST) imaging method for nontransparent 3D samples
Source: BMC Biol. 2019 Jan 7;17:1. doi: 10.1186/s12915-018-0614-4 (PMC6323867; doi:10.1186/s12915-018-0614-4)
Supplement: Supplementary file 20 — Table S1. Table describing the imaging conditions per Figure. (PDF 354 kb) [file 12915_2018_614_MOESM20_ESM.pdf]

Table S1: Imaging conditions per Figure.

Figure	Object	Pixelsize X(µm)	Y*(µm)	Z(µm)	No. of pixels	Rotation step angle (deg)
2	Resistor	37.91	37.91	37.91	512 x 512	0.9
	Metasequoia seed cone	37.91	37.91	37.91	512 x 512	0.9
3	Lego figures µCT	51.67	51.67	51.67	672 x 564	0.7
	Lego figures ALMOST	18.96	18.96	18.96	1024 x 1024 but downscaled to 512 x 512	0.45
4	Fly red eyes	4.2	4.2	4.2	1024 x 1024	0.45
	Fly GlaBC/CyO	4.2	4.2	4.2	1024 x 1024	0.9
5	Living Xenopus	6.3	6.3	6.3	512 x 512	0.9
	Spinning disk Xenopus	1.08	1.08	2.5	1002 x 1004	/
Sup. 3	Rosemary beetle	18.35	18.35	18.35	512 x 512	0.9
	Coin 1 cent	37.91	37.91	37.91	512 x 512	0.9
Sup. 4	Grid 300	4.3	4.3	4.3	1024 x 1024	0.45
	Grid 400	4.3	4.3	4.3	1024 x 1024	0.45
	Grid 17	4.2	4.2	4.3	1024 x 1024	0.45
	Dyna beads	4.2	4.2	4.2	1024 x 1024	0.45
Sup. 5	Shell	37.91	37.91	37.91	512 x 512	0.9
Sup. 6	Drosophila larvae	11.09	11.09	11.09	512 x 512	0.9
Sup. 7	Fixed Xenopus	6.2	6.2	6.2	512 x 512	0.9

***y is the rotational axis in the case of tomographic imaging procedures.**
