# Supplementary material for: A Label-free Multicolor Optical Surface Tomography (ALMOST) imaging method for nontransparent 3D samples
Source: BMC Biol. 2019 Jan 7;17:1. doi: 10.1186/s12915-018-0614-4 (PMC6323867; doi:10.1186/s12915-018-0614-4)
Supplement: Supplementary file 21 — Table S2. Table containing details about the visualization. (PDF 343 kb) [file 12915_2018_614_MOESM21_ESM.pdf]

Table S2 visualization used.

Figure	Channels imaged (ALMOST, unless indicated otherwise)	Render mode	Colors used (Arivis)	Invert
Yellow resistor	Red, green & blue filter	Max. intensity	Red, green & blue	Invert box ticked
Green resistor	Color camera (red, green & blue)	Max. intensity & volumetric	Red, green & blue<br>Cyan, magenta & yellow	Invert box ticked
Metasequoia	Red, green & blue filter	Volumetric	Red, green & blue	Mirrored cubic
Rosemary beetle	Red, green & blue filter	Volumetric	Cyan, magenta & yellow	Invert box ticked
Coin	Red, green & blue filter	Volumetric	Cyan, magenta & yellow	Invert box ticked
Lego	Red, green & blue filter	Max. intensity	Red, green & blue	Negative linear (workman) & mirrored cubic (Dalton)
400 mesh grid	No filter & transmitted light OPT	Volumetric	Red & blue	/
17µm bar grid	No filter	Volumetric	Grey	/
Zoom fly eye	No filter	Volumetric	Grey	Invert box ticked
Shell	6 filters: 337/50, 420/40, 460/50, 525/50, 600/50, 690/70	Volumetric	Wavelength: 377, 420, 460, 525, 600 & 690	Invert box ticked
Drosophila larvae	No filter	Volumetric	Grey	Invert box ticked
Fly fluo eyes	Red, green & blue filter & green fluorescence OPT	Volumetric	Cyan, magenta & yellow	Invert box ticked & fluorescence not inverted
Fly head CD & GlaBC/Cyo	Red, green & blue filter	Volumetric	Cyan, magenta & yellow	Invert box ticked
Xenopus fixed	No filter	Volumetric	Grey	Invert box ticked
Xenopus live	No filter	Volumetric	Grey	Invert box ticked
Xenopus fixed older stage	No filter & transmitted light OPT & UV fluorescence OPT	Visualized in Imaris:	Visualized in Imaris:<br>Green & magenta & cyan	/
LED	No filter	Volumetric	Gradient: blue green yellow red	/
